# Effects of midazolam, pentobarbital and ketamine on the mRNA expression of ion channels in a model organism *Daphnia pulex*

**DOI:** 10.1186/1471-2253-13-32

**Published:** 2013-10-18

**Authors:** Changhong Dong, Anmin Hu, Yang Ni, Yunxia Zuo, Guo Hua Li

**Affiliations:** 1 Laboratory of Anesthesiology and Critical Care Medicine, Translational Neuroscience Center, West China Hospital, Sichuan University, Chengdu 610041, China; 2 Department of Anesthesiology, West China Hospital, Sichuan University, Chengdu, China

**Keywords:** Midazolam, Pentobarbital, Ketamine, mRNA, Ion channel, Daphnia pulex

## Abstract

**Background:**

Over the last few decades intensive studies have been carried out on the molecular targets mediating general anesthesia as well as the effects of general anesthetics. The γ-aminobutyric acid type A receptor (GABA_A_R) has been indicated as the primary target of general anaesthetics such as propofol, etomidate and isoflurane, and sedating drugs including benzodiazepines and barbiturates. The GABA_A_R is also involved in drug tolerance and dependence. However, the involvement of other ion channels is possible.

**Methods:**

Using reverse transcription and quantitative PCR techniques, we systematically investigated changes in the mRNA levels of ion channel genes in response to exposure to midazolam, pentobarbital and ketamine in a freshwater model animal, *Daphnia pulex*. To retrieve the sequences of *Daphnia* ion channel genes, Blast searches were performed based on known human or *Drosophila* ion channel genes. Retrieved sequences were clustered with the maximum-likelihood method. To quantify changes in gene expression after the drug treatments for 4 hours, total RNA was extracted and reverse transcribed into cDNA and then amplified using quantitative PCR.

**Results:**

A total of 108 ion channel transcripts were examined, and 19, 11 and 11 of them are affected by midazolam (100 μM), pentobarbital (200 μM) and ketamine (100 μM), respectively, covering a wide variety of ion channel types. There is some degree of overlap with midazolam- and pentobarbital-induced changes in the mRNA expression profiles, but ketamine causes distinct changes in gene expression pattern.

In addition, flumazenil (10 μM) eliminates the effect of midazolam on the mRNA expression of the GABA_A_ receptor subunit Rdl, suggesting a direct interaction between midazolam and GABA_A_ receptors.

**Conclusions:**

Recent research using high throughput technology suggests that changes in mRNA expression correlate with delayed protein expression. Therefore, the mRNA profile changes in our study may reflect the molecular targets not only in drug actions, but also in chronic drug addiction. Our data also suggest the possibility that hypnotic/anesthetic drugs are capable of altering the functions of the nervous system, as well as those non-nerve tissues with abundant ion channel expressions.

## Background

Midazolam is a benzodiazepine and widely used as an anxiolytic, anticonvulsant, sleep aid, muscle relaxant, and antipsychotic. Pentobarbital is a short-acting barbiturate that is used as a sedative and anesthetic agent. Like other barbiturates, pentobarbital produces a wide spectrum of dose-dependent effects, including sedation, hypnosis, anesthesia and finally coma. Although their use has decreased over the years because of high abuse potential, barbiturates are still being prescribed to many patients, such as epilepsy patients and people with sleep disorders. The principal mechanism of actions of benzodiazepines and barbiturates is believed to be positive allosteric modulation of the γ-aminobutyric acid (GABA) type A receptor (GABA_A_R) [1-3]. Ketamine, a rapid acting anesthetic agent and a popular drug of abuse, has diverse effects, including antidepressant action and analgesic effects on chronic pain [4]. These actions were traditionally believed to arise from the inhibition of NMDA receptors. However, NMDAR blockers, such as MK-801, fail to mimic the actions of ketamine [5,6]. Furthermore, knockout of NR2A subunit reduces but does not eliminate the actions of ketamine [7-9]. Ketamine has also been shown to positively modulate the function of the cerebellar GABA_A_ receptors containing α6 and δ subunits [10].

Chronic use of midazolam, phenobarbital and ketamine produces tolerance and physical dependence. The homeostatic theory of drug tolerance [11] claims in a modern form that the functional tolerance results from altered function or expression of proteins in a way to reduce the effects of the drugs. Based on this theory, for example, the GABA_A_R, which is positively modulated by drugs such as benzodiazepines and barbiturates, is expected to be downregulated after prolonged or repetitive drug exposure. This adaptive response may involve, although not necessarily, reduction in the mRNA levels of the GABA_A_R subunits. Therefore, comparison of changes in mRNA expression patterns in response to different drugs may offer clues to molecular targets involved in drug actions and drug tolerance/dependence.

The effects of benzodiazepines and barbiturates on the mRNA expression of different GABA_A_R subunits have been investigated over the last few decades [12-17]. Studies of the transcriptional responses of the NMDAR genes to diazepam [18-20] and flurazepam [21] are also available. Unfortunately, such studies have led to conflicting results. For example, diazepam was found to downregulate GABA_A_R α1 mRNA expression in the cerebral cortex by several research groups [12-14], but others [17,22] found diazepam ineffective. Similar situations were found for other GABA_A_R subunits [23]. Diazepam was found to increase the cortical mRNA contents of NR1 and NR2B subunits [18-20]. However, another study showed decreases in hippocampal NR2B mRNA and protein after chronic flurazepam treatment [21]. Compared with the GABA_A_R and NMDAR, the effects of barbiturates, benzodiazepines and ketamine on the mRNA expression of other types of ion channels are largely unknown.

Ion channels are complex proteins forming ion-per-meable pathways through biological membranes. The ion channels tested in this study include the P-domain channels [24], the pentameric ligand-gated ion channels (pLGICs), the ENaC/Deg ion channels, the ATP-gated ion channels (P2X receptors), the calcium release-activated calcium (CRAC) channels, the inositol 1, 4, 5-trisphosphate receptors (IP3Rs) and ryanodine receptors (RyRs), and the chloride ion channels. The only known ion channel genes not included in this study were the invertebrate ionotropic receptors (IRs), a variant subfamily of iGluRs [25,26].

The P-domain channels have a common pore architecture composed of four homologous pore-domains contributed by one, two or four subunits arranged in four-fold symmetry. This group of channels include voltage-gated potassium (Kv) channels, cyclic nucleotide-gated (CNG) channels, hyperpolarization-activated CNG (HCN) channels, voltage-gated calcium (CaV) channels, voltage-gated sodium (NaV) channels, sodium-leak channels (NALCN), two-pore channels (TPCs), transient receptor potential (TRP) channels, and glutamate-gated ion channels (or ionotropic glutamate receptors, iGluRs).

Most pLGICs are gated by extracellular ligands, and include the nicotinic acetylcholine receptors (nAChRs), 5-hydroxytryptamine type 3 receptors (5-HT3Rs) GABA_A_Rs and glycine receptors (GlyRs). The mammalian pLGIC superfamily also includes zinc-activated ion channels (ZACNs), the invertebrate pLGIC superfamily also includes the glutamate-gated chloride (GluCl) channels, histamine-gated chloride (HisCl) channels, and pH-sensitive chloride (pHCl) channels.

Both ENaC/Deg channels and P2X receptors are trimeric and share similar transmembrane topology. However, they are not homologous in amino acid sequences. The CRAC channels are hexameric plasma membrane proteins mediating the entry of extracellular Ca^2+^ when the intracellular Ca^2+^ stores are depleted. The IP3Rs and RyRs, on the other hand, are intracellular membrane proteins important to intracellular Ca^2+^ signaling. The chloride channels are anion permeable protein complexes (excluding the GABA_A_R and GlyR) and are less-well understood.

The aim of our study is to measure the effects of midazolam, pentobarbital and ketamine on the ion channel mRNA expression in the water flea *Daphnia pulex*, a freshwater crustacean with great potential for biomedical research (http://www.nih.gov/science/models/).

## Methods

### *Daphnia* cultures and treatments

**Figure 1:**
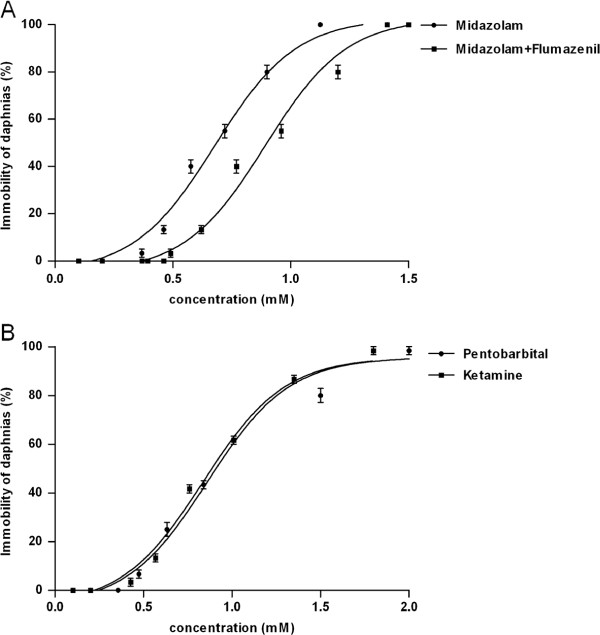
**Daphnia immobility dose responses of midazolam and midazolam + flumazenil (Panel A), pentobarbital and ketamine (Panel B).** 20 daphnids were treated with midazolam, midazolam + flumazenil, pentobarbital and ketamine at series of concentrations. The numbers of immobilized daphnids were observed 4 hours after each treatment. The immobile ratio were presented as means ± SE (n = 3).

The method for culturing *D. pulex* was the same as previously described [27]. Briefly, daphnids from a single clone were kept in a temperature- and photocycle-controlled tank (20 ± 1°C, 16:8 h light-dark cycle) and fed daily with a mixture of *Saccharomycetes* and *Spirulina*. One-fifth of the medium was renewed every second day. Midazolam, pentobarbital sodium and ketamine stock solutions of 10, 100 and 100 mM, respectively, were made in distilled water, and diluted to final concentrations before use. Since the constant jumping movements of *Daphnia* may complicate experimental results if the animals are immobilized by drugs while the animals in the control group are free to swim, drugs of subanesthetic concentrations based on the EC50 values (Figure 1) were used to investigate the transcriptional responses of ion channel genes. For this purpose, daphnids were exposed to midazolam (100 μM), pentobarbital (200 μM), and ketamine (100 μM) for 4 hours, and killed by crushing immediately for mRNA quantification by RT-qPCR. Protocols were approved by the Scientific and ethics committee of Sichuan university and adhered to the international guidelines for animal care.

### Sequence analysis and phylogenetic inference

The *Daphnia* genome (http://wfleabase.org/), NCBI and Uniprot protein database were searched for the *Daphnia* ion channel gene models based on known human or *Drosophila* genes. Sequences were analyzed with the maximum-likelihood (ML) method using MEGA 5.10 program [28]. The sequences used are designated in succession by the abbreviation of the species (Hs for *Homo sapiens*, Dm for *Drosophila melanogaster,* Ce for *C. elegans*, Am for *Apis mellifera*, and Dpul for *Daphnia pulex*) and the gene name.

### RNA extraction, reverse transcription and polymerase chain reaction (PCR)

Total RNA extraction, reverse transcription and PCR were performed as previously described [27]. Briefly, RNA was harvested from 50 crushed daphnids for phase separation, precipitation, and quantification. cDNA was generated using PrimeScriptTM RT reagent Kit DRR037A (TaKaRa), and amplified first by regular PCR to screen primers, which were designed using Primer 3 software [29] based on the scaffold sequences (http://wfleabase.org/). Successful primers (Additional file 1: Table S1) were then used for qPCR to quantify changes in gene expression after drug treatment. qPCR was carried out on an iQ5 system (Bio-Rad) using SYBR Premix Ex TaqTM II KIT DRR081A (TaKaRa). Each reaction was run in triplicate and contained 2 μl of cDNA template along with 0.8 μM primers in a total volume of 20 μl. Cycling parameters were 95°C for 30 s to active the DNA polymerase, then 40 cycles of 95°C for 5 s, 55°C for 30 s and 72°C for 30 s. Melting-curves were performed to verify only a single product without primer-dimers. Data were normalized against a house-keeping reference gene β-actin, and were analyzed using the 2^-ΔΔCT^ method [30].

### Statistical analysis

All data were presented as means ± SE. qPCR data represent the average of 5 replicate experiments; all results were normalized to β-actin, an internal control, and then to control group. Differences in relative expression of genes were assessed using paired-*t* test (*n* = 5). Statistical significance was set at a level of *P* < 0.05 (*) and *P* < 0.01 (**).

## Results

### Phylogenetic analysis

More than 120 gene models of ion channel proteins have been predicted in the *Daphnia* genome and 108 of them were successfully amplified by RT-PCR (Additional file 2: Table S2). For comparison purposes, these proteins are classified into different categories based on sequence homology molecular structure, and ion selectivity in the case of chloride channels.

### The four-fold symmetric P-domain ion channels

A total of 53 P-domain channel genes are detected at transcript level in *Daphnia* (Additional file 2: Table S2), and they are classified into four superfamilies: Kv/CNG, CaV/NaV, TRP and iGluR. The Kv/CGN members are further classified into three families based on subunit transmembrane topology: the two transmembrane-helix (2TM) family (Additional file 3: Figure S1), the 6TM family (Additional file 4: Figure S2), and the 4TM (K2P) family (Additional file 5: Figure S3). The 4TM potassium genes resulted from duplication of 2TM genes during the evolution, thus the name K2P, while the 6TM domain consists of a 2TM domain and a voltage sensor domain. The CNG and HCN channels are homologous to the 6TM Kv channels (Additional file 4: Figure S2). The CaV or NaV channel contains a single principal subunit with four 6TM domains. The NALCN and TPC channels also belong to the NaV/CaV superfamily. The NALCN is a voltage-independent, TTX-insensitive, and nonselective cation channel underlining the background Na^+^ leak current [31]. TPC channels are intracellular ion channels mediating the second messenger NAADP-regulated Ca^2+^ release. The *Daphnia* NaV/CaV superfamily contains 7 members: 3 CaV, 2 NaV, 1 NALCN and 1 TPC genes (Additional file 6: Figure S4).

TRP channels are highly diverse in function, structure and distribution, with 28 mammalian TRP genes classified into six subfamilies: TRPC, TRPV, TRPM, TRPML, TRPA and TRPP. In addition, the invertebrates have a group known as TRPN, which is also found in zebrafish. The *Daphnia* genome contains 13 TRP subunit genes (Additional file 7: Figure S5).

The iGluRs are tetrameric and can be divided into three subfamilies based on pharmacology and homology: AMPA, NMDA and kainate (KA) receptors. The *Daphnia* genome contains eight iGluR homologs: Dpul_Glu-RI, Dpul_Nmdar1-3, Dpul_KaiR1-4 (Additional file 8: Figure S6). A variant iGluR subfamily, the ionotropic receptors (IRs), was not included in this study.

### The pentameric ligand-gated ion channels

The *Daphnia* genome contains 20 pLGIC genes: 12 nAChR (Additional file 9: Figure S7), 5 GABA_A_R (Grd, Rdl, RdlL, Lcch3 and CG8916), 1 GluCl, and 2 HisCl genes (Additional file 10: Figure S8). There is no RdlL counterpart in *Drosophila*. The classification of the *Daphnia* pLGICs into nAChR and GABA_A_R groups is simply based on homology.

### The ENaC/Deg channels and P2X receptors

The ENaC/Deg channels and P2X receptors are distinct classes of trimeric protein complexes. The *Daphnia* ENaC/Deg homologs are quite diverse, with 14 members detected at the transcript level (Additional file 11: Figure S9). Two *Daphnia* P2X genes are detected at the transcript level (Dpul_P2XL1 and Dpul_P2XL2 (Additional file 12: Figure S10).

### The CRAC channels, IP3Rs, RyRs and the chloride channels

The *Daphnia* genome contains one CRAC channel gene, one IP3R gene, and one RyR gene (Additional file 12: Figure S10). There are at least five distinct classes of Cl^–^ channels, including the ClC channels, the CLIC proteins, bestrophin, the tweety chloride channels, and anoctamin/TMEM16. The *Daphnia* genome contains 7 ClC genes, 2 CLIC genes, 4 bestrophin genes, 1 tty gene and 3 TMEM16 genes (Additional file 13: Figure S11).

### Dose-dependent immobility upon midazolam, pentobarbital, and ketamine treatment

In order to determine the subanesthetic concentrations for chronic treatment in our mRNA assay, daphnids were exposed to aquarium water containing a series of concentrations of midazolam, pentobarbital, or ketamine for four hours (Figure 1). Midazolam, pentobarbital and ketamine immobilized the daphnids at EC50 values of 0.65, 0.92 and 0.84 mM, respectively. Flumazenil (10 μM), a competitive antagonist of benzodiazepines and used to reverse the actions of benzodiazepines in clinical settings, shifted the dose-response curve of midazolam to the right (EC50 = 0.86 mM , Figure 1A), but had no effects of its own on the immobility of the daphnids up to 100 μM. Interestingly, ketamine at concentrations of >200 μM produced a consistent circling behavioral phenotype, mimicking the core behavior aspects of rodents and fish administered with ketamine [32,33]. This aberrant behavior was completely absent in the control daphnids. Ketamine-induced immobility started at higher concentration (~400 μM). Based on these dose-dependent responses, we exposed daphnias to 100-μM midazolam, 200-μM pentobarbital and 100-μM ketamine for 4 hours for our mRNA assays. At these concentrations, even longer treatment (10 hours) did not result in death of the animals. The use of subanesthetic concentrations instead of anesthetic ones were due to the fact that daphnids jump constantly and this behavior may complicate the experimental results if the animals were immobilized by drugs while the animals in the drug-free (control) group were free to swim.

### Effects of midazolam on the transcription of *daphnia* ion channel genes

**Figure 2:**
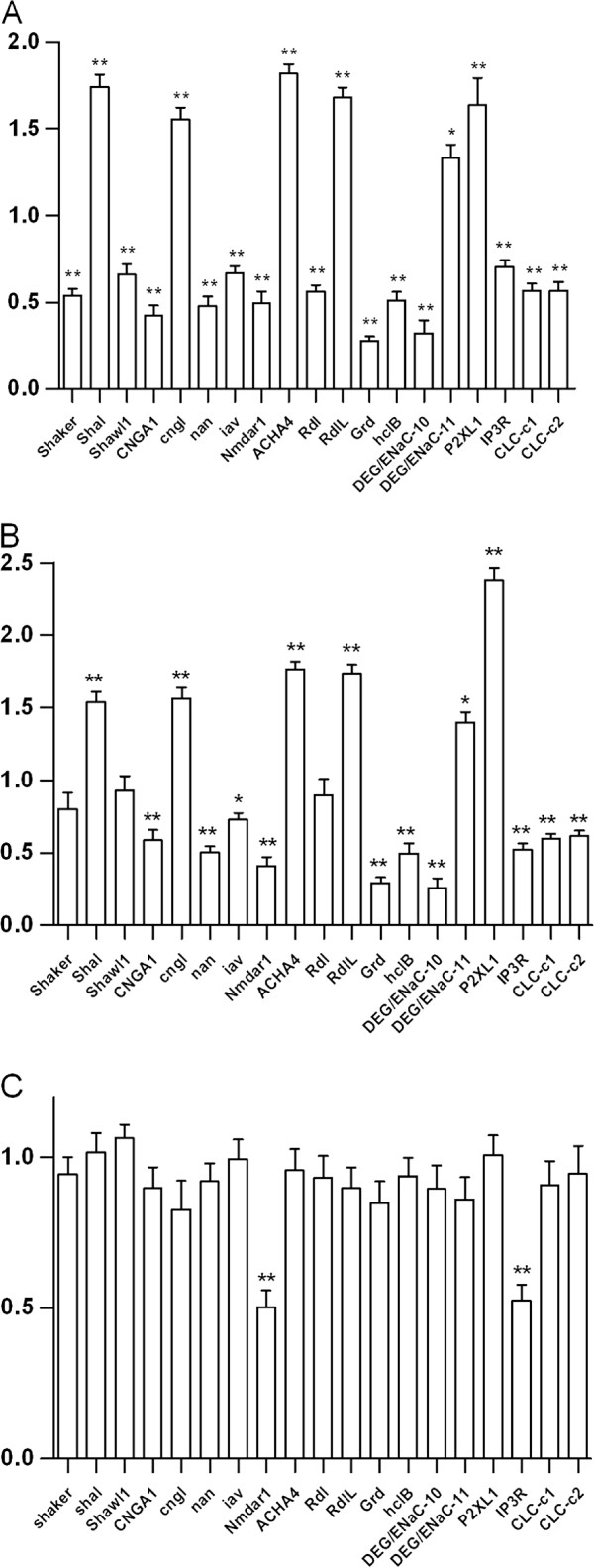
**Effects of midazolam and flumazenil on the expression of *Daphnia *ion channel genes. (A)**. Daphnids were exposed to aquarium water containing 100 μM midazolam for 4 hours and killed for mRNA quantification. Out of 108 ion channel genes, 19 genes are affected by midazolam. **(B)**. Daphnids were co-treated with flumazenil (10 μM) along with midazolam (100 μM) for 4 hours and killed for mRNA quantification. Flumazenil abolishes the effects of midazolam on the mRNA expression of Rdl, Shawl1 and Shaker. **(C)** Daphnids were exposed to 10 μM flumazenil alone for 4 hours before mRNA quantification. The Nmdar1 and IP3R transcripts were significantly reduced by flumazenil. Statistical significance was set at a level of *P* < 0.05 (*) and *P* < 0.01 (**).

Out of the 108 genes tested, the transcription of 6 genes was upregulated by midazolam treatment, including the 6TM Kv channel shal, the CNG channel cngl, the nAChR subunit ACHA4, the GABA_A_R RdlL, DEG/ENaC-11 and P2XL1 (Figure 2). Meanwhile, midazolam downregulated transcription of 13 genes, including the 6TM Kv channels shaker and shawl1, the CNG channel CNGA1, the TRP channels nan and iav, the NMDA receptor Nmdar1, the GABA_A_R Rdl and GRD, the HisCl hclB, the CLC Clc-c1, Clc-c2, DEG/ENaC-10 and IP3R (Figure 2A). Flumazenil (10 μM) abolished the effects of midazolam on the mRNA expression of Rdl, Shawl1 and Shaker (Figure 2B). Flumazenil alone, however, did not affect the Rdl, Shawl1 and Shaker mRNA expression. In addition, we found that flumazenil (10 μM) alone downregulated Nmdar1 and IP3R transcription (Figure 2C).

### Effects of pentobarbital on the transcription of *daphnia* ion channel genes

**Figure 3:**
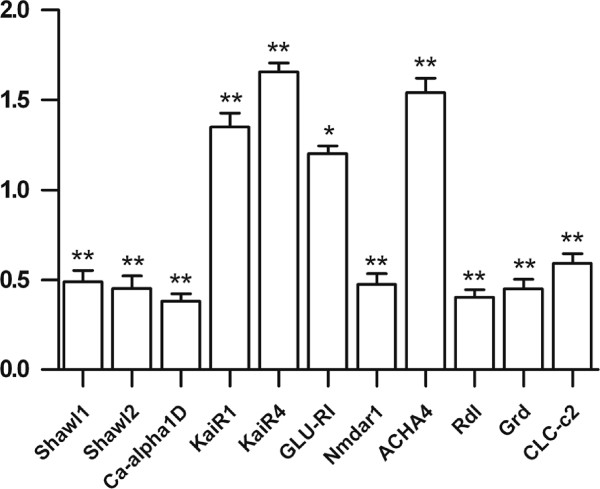
**Effects of pentobarbital on the expression of the *Daphnia *ion channel genes.** Daphnids were exposed to 200 μM pentobarbital for 4 hours and killed for mRNA quantification. Seven genes are downregulated and four genes are upregulated at transcriptional level by pentobarbital. Statistical significance was set at a level of *P* < 0.05 (*) and *P* < 0.01 (**).

Pentobarbital (200 μM) downregulated the expression of the 6TM Kv channel genes Shawl1 and Shawl2, the CaV channel gene Ca-alpha1D, the NMDAR Nmdar1, the GABA_A_Rs Rdl and GRD, the CLC Clc-c2, while the KAR gene KaiR1 and KaiR4, the AMPAR gene Glu-RI and the nAChR ACHA4 were upregulated by pentobarbital (Figure 3).

### Effects of ketamine on the transcription of *daphnia* ion channel genes

**Figure 4:**
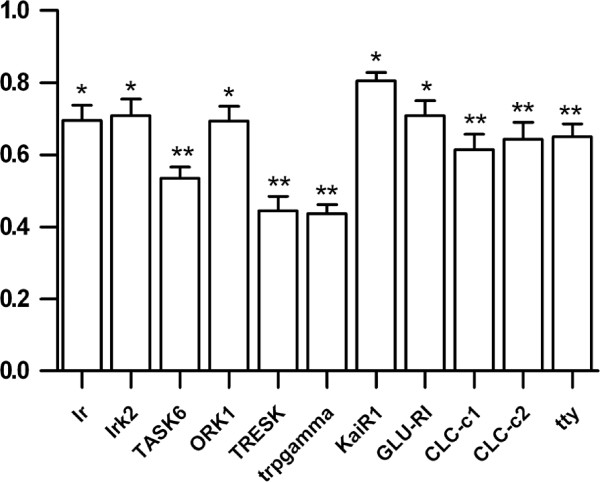
**Effects of ketamine on the expression of Daphnia ion channel genes.** Daphnids were exposed to 100 μM ketamine for 4 hours and killed for mRNA quantification. Among 108 ion channel genes, ketamine affects 11 genes at transcriptional level. Surprisingly, all of the 11 genes are downregulated by ketamine. Statistical significance was set at a level of *P* < 0.05 (*) and *P* < 0.01 (**).

Ketamine (100 μM) downregulated transcription of 11 genes, including the 2TM Kir channels Ir and Irk2, the K2P channels Task6, Ork1 and TRESK, the TRP channel Trpgamma, the iGluRs Glu-RI and KaiR1, the CLC CLC-c1 and CLC-c2, and tty (Figure 4).

## Discussion

Invertebrate model organisms, such as *C. elegans*[34] and *Drosophila*[35], are desirable models for the studies of the mechanisms of drug tolerance/dependence. These simple animals share many complex traits with mammals, even at behavioral levels [36]. Furthermore, the major groups of ion channels are conserved, simplifying the dissection of core molecular machinery responsible for drug addiction.

The choice of midazolam concentration (100 μM) for the mRNA assays was based on the dose-response curve (Figure 1). Compared to clinic concentrations, which are around 1 μM [37], the high midazolam concentration may result in nonspecific effects of the drug and consequently in changes in gene expression that are not related to midazolam action on GABA_A_ receptors. Nonetheless, the down-regulation of the Rdl mRNA by midazolam appears to be directly mediated by GABA_A_ receptors, since this effect is eliminated by flumazenil (10 μM), a competitive antagonist of benzodiazepines. In addition, the direction of Rdl mRNA regulation is consistent with the prediction by the homeostasis theory of drug tolerance [11]. Despite the role of mRNA regulation is not explicitly implied in the theory, recent studies suggest that protein and mRNA expression levels are correlated [38-40]. Interestingly, flumazenil also eliminates midazolam-induced reduction in the mRNA levels of Shawl1 and Shaker (Figure 2B), suggesting a possibility of crosstalk between GABA_A_R and potassium channel signalings.

The short time course of drug treatment in the current study may undermine the relevance of the results to questions of addiction and drug dependence. For the development of tolerance and dependence in mammals, chronic treatments lasting weeks to months are required. In *Drosophila*, the lifespan of which is around 30 days under common culture conditions, the time courses of chronic treatments are much shorter, but vary greatly depending on experiment scenarios and the purposes of the study. For example, induction of neurodegeneration mimicking Parkinson's disease requires days of treatments [41,42]. Alcohol addiction in flies, on the other hand, develops more rapidly. Two types of addiction have been identified [35]: rapid tolerance can be induced by a single brief (less than 60 min) exposure to ethanol, while chronic tolerance requires prolonged (~24 h) ones. Chronic alcohol tolerance depends on protein synthesis, while molecular events downstream of protein synthesis contribute to acute tolerance. However, in general the emerging picture of the regulation of mRNA and protein expression is perhaps more complex than initially thought [43]. Accumulating evidence shows that mRNA expression correlate best with delayed protein expression. For example, the mRNA abundance changes in yeasts occur in a time window of 20 to 240 min after rapamycin treatment, while the protein abundance changes mostly occur at 4 and 6 h of the treatment [44]. This temporal correlation between mRNA and protein expression is not specific to rapamycin, since oxidative stress also induces rapid transcriptional changes which peak at 60 min after the treatment and decay quickly, while the protein expression response is much slower [38]. Nothing is known about the temporal relationship between mRNA and protein abundances in the development of chronic drug tolerance. However, it is highly possible in our case that changes in ion channel mRNA levels may lead to delayed alteration in protein abundance and chronic response to the drug treatments.

Our results suggest that *Daphnia* are feasible model animals for the investigation of the role of GABA_A_ receptors in drug addiction, including addiction to benzodiazepines. Many early studies suggest that insect GABA_A_ receptors were relatively insensitive to benzodiazepines. These studies involved the use of recombinantly expressed GABA_A_ receptors, such as the homo-oligomeric Rdl receptor [45,46] and the Grd/LCCH3 receptor [47]. There is, however, evidence that native insect GABA_A_ receptors are modulated by some benzodiazepines. For example, Lees et al. (1987) [48] showed that the 7-nitro benzodiazepine flunitrazepam enhanced the amplitude of GABA-induced currents by up to 70%. More recently, Buckingham et al. (2009) [49] observed that GABA-induced currents in acutely dissociated insect motor neurons were enhanced by both Ro5-4864 and diazepam, whereas clonazepam was ineffective. The pharmacological differences between recombinant and native GABA_A_ receptors suggest more complex subunit combinations in native GABA_A_ receptors. The involvement of GABA_A_ receptors in benzodiazepine dependence appears to be evolutionarily conserved. It is observed in flatworms, the simplest bilaterian animals, that flumazenil (10 μM) antagonizes the abstinence-induced withdrawal from midazolam (10 μM), but has no effect of its own on the behavior of the animals [50].

In *Daphnia*, ketamine at lower concentrations produces increased movements in the form of circling swimming, while immobility starts at higher concentrations. The dissociation of hyperactivity from immobility is interesting and raises the possibility that the molecular targets responsible for ketamine-induced hyperactivity are different from those for immobility. Currently, we found that ketamine-induced changes in the mRNA expression profile are quite different from those seen with midazolam and pentobarbital treatments. It is worth noting that most of the *Daphnia* genes affected by ketamine are from the P-domain channel group, including the 2TM (Ir, Irk2), 4TM (Task6, ork1, TRESK), TRP (Trp-gamma), iGluR (Glu-R1, KaiR1) families. A P-domain is basically the 2TM domain and composed of two transmembrane helices connected by a loop region referred to as the P-loop. Four P-loops in a ion channel form the pore selectivity filter. It is believed that the open-channel blockers of NMDARs, such as MK-801 and ketamine, bind to the P-loop amino acid residues. It is therefore highly possible that ketamine may interact with the other P-domain channels in a similar way. The K2P channel, ork1, has been implicated in the regulation of cardiac automatic activity in *Drosophila*[51], as well as in mammals [52]. In mammals, the kainate receptors are implicated in chronic pain regulation [53]. The *Daphnia* KaiR1 gene is homologous to the mammalian KARs and was downregulated at the transcript level. Unfortunately, data regarding the physiology of KaiR1 is unavailable.

Compared with the P-domain channels and pLGICs, other ion channels are less well understood. Nonetheless, recent studies suggest that the CLC chloride channels play a role in synaptic transmission and plasticity [54,55], as well as in neuronal excitability [56]. We found in *Daphnia* that the Clc-c2 mRNA expression was downregulated by midazolam, pentobarbital and ketamine, while the CLC-c1 mRNA expression was also downregulated by midazolam and ketamine, suggesting a new type of molecular targets possibly involved in drug addiction.

## Conclusions

The major groups of ion channels are highly conserved across the animal kingdom. In addition, simple animals, such as *C. elegans* and *Drosophila*, share many complex traits with mammals, even at behavioral levels [36]. Therefore, the use of invertebrate model organisms may greatly simplify the dissection of core molecular machinery responsible for drug actions and addiction. In *Daphnia*, midazolam, pentobarbital and ketamine cause distinct mRNA expression profiles and this observation provide insights into potential novel molecular targets involved in drug actions and addiction.

## Competing interests

The authors declare that they have no competing interest.

## Authors’ contributions

CD carried out the pharmacological and qPCR assays, performed the statistical analysis and helped draft the manuscript. AH carried out sequence analysis and screened the primers. YN was involved in the pharmacological studies. YZ contributed to the study design and helped draft manuscript. GHL designed the study and drafted the manuscript. All authors read and approved the final manuscript.

## Pre-publication history

The pre-publication history for this paper can be accessed here:

http://www.biomedcentral.com/1471-2253/13/32/prepub

## Supplementary Material

Additional file 1: Table S1 Gene name, Gene ID, Genome Map, Primer, product size and qPCR efficiency. The forward and reverse primers, gene ID, product size and qPCR efficiency for indicated genes were given, based on the scaffold sequences of the *Daphnia* Genomics Consortium. All primers were designed using Primer 3 (version 0.4.0). The expression and length of each gene were confirmed by reverse transcription PCR. qPCR efficiency of target genes was calculated by Bio-Rad iQ5 software. Click here for file

Additional file 2: Table S2 Gene name, synonyms of the gene name and Uniprot accession numbers used in phylogenies. Click here for file

Additional file 3: Figure S1 Phylogenetic tree of the 2TM-K_V_ (Kir) channels. The Kir subunit possesses a P-domain composed of two transmembrane helices connected by a P-loop. Four Kir subunits assemble to for a channel. The mammalian Kir family comprises the classical inward-rectifying K^+^ channels (Kir2), the K_ATP_ channels (Kir6), G protein-activated K^+^ channels (Kir3), and K^+^-transport channels. The invertebrate Kir genes form distinct clusters. The *Daphnia* genome contains two Kir genes: Dpul_Ir and Dpul_Irk2. The homolog of the *Drosophila* Irk3 is absent in *Daphnia* genome. Abbreviation: Hs, *Homo sapiens*; Dm, *Drosophila melanogaster*; Ce, *Caenorhabditis elegans*; Dpul, *Daphnia pulex*. Click here for file

Additional file 4: Figure S2 Phylogenetic tree of the 6TM-Kv, CNG and HCN channels. The 6TM-Kv channels are highly diverse in sequence, structure and function. In addition to the P-domain, each 6TM-K_V_ subunit obtains a voltage sensor domain (VSD) composed of four transmembrane helices. The CNG and HCN channels, although not classified as Kv channels, are homologous to the KCNH K_V_ channels. *Daphnia* and *Drosophila* share similar gene sets for the 6TM-K_V_/CNG/HCN channel group, with subtle differences. The homolog of the *Drosophila* elk is absent in the *Daphnia* genome. *Daphnia* have two homologs of the *Drosophila* Shawl genes, namely Shawl1 and Shawl2, while *Drosophila* have one. Click here for file

Additional file 5: Figure S3 Phylogenetic tree of the 4TM-K_V_ (K2P) channels. The K2P subunit consists of two P-domains, and two such subunits assemble to form a channel. *Daphnia* have five K2P members: Dpul_TWIK, Dpul_Task6, Dpul_Task7, Dpul_Ork1 and Dpul_TRESK. The *Daphnia* homolog of the mammalian TWIK is detected at transcript level, but the *Drosophila* counterpart is absent. As in *Drosophila*, the TREK member of the K2P channels is also absent in *Daphnia*. Abbreviation: Hs, *Homo sapiens*; Dm, *Drosophila melanogaster*; Ce, *Caenorhabditis elegans*; Dpul, *Daphnia pulex*. Click here for file

Additional file 6: Figure S4 Phylogenetic tree of the CaV, NaV, NALCN and TPC channels. The Ca_V_ and Na_V_ channels are thought to arise from the potassium channels during evolution. The CaV/NaV channel contains a single principal (α1) subunit with four 6TM domains. The human genome contains ten α1 genes, which can be further clustered in three groups: CaV1 (L-type), CaV2 (P/Q, N, and R type), and CaV3 (T-type). The *Daphnia* genome predicts three genes: Dpul_Ca-alpha1D (CaV1), Dpul_Ca-alpha1T (CaV2) and Dpul_cac (CaV3). NALCN is represented by a single gene in human, *Daphnia* and *Drosophila*. Unlike the CaV, NaV and NALCN, the TPC subunit contains two P-domains and assembles as a dimer. The transcript of the TPC encoding gene is detected in *Daphnia*. TPC Gene models are also available for many insects, such as *Apis mellifera*, but absent in *Drosophila*. Abbreviation: Hs, Homo sapiens; Dm, *Drosophila melanogaster*; Am, *Apis mellifera*; *Caenorhabditis elegans*; Dpul, *Daphnia pulex*. Click here for file

Additional file 7: Figure S5 Phylogenetic tree of the TRP channels. Despite of the structure and function diversities, all TRP subunits have a common design for the transmembrane domain, which basically is a 6TM domain. So far 28 mammalian TRP subunit encoding genes are known and belong to six subfamilies known as the TRPC, TRPV, TRPM, TRPML, TRPP and the TRPA, and all these groups have invertebrate orthologs. The *Daphnia* genome predicts 13 TRP subunit genes and all were detected at transcript level, including a 3 TRPC-like genes (Dpul_TRP, Dpul_TRPL and Dpul_Trpgamma), 2 TRPM-like gene (Dpul_TRPM1, Dpul_TRPM2), 4 TRPA-like genes (Dpul_TRPA5, Dpul_pain, Dpul_pyx1, Dpul_pyx2), 3TRPV genes (Dpul_nan, Dpul_iav, TRPV_nompc), 1TRPML gene (TRPML). TRPP genes are absent in *Daphnia*. Abbreviation: Hs, Homo sapiens; Dm, *Drosophila melanogaster*; Ce, *Caenorhabditis elegans*; Am, *Apis mellifera*; Dr, *Danio rerio*; Dpul, *Daphnia pulex*. Click here for file

Additional file 8: Figure S6 Phylogenetic tree of the iGluRs. The iGluR is a heterotetramer containing four P-domains, each in one subunit. The mammalian iGluRs are divided into three groups: AMPA, NMDA and KA receptors. There are two other classes with respect to sequence similarity known as the delta class (GRID) and the kainate-binding proteins (KBP). The invertebrate iGluRs display considerable variations, especially in the KAR branch. In addition, a GRIN3-like gene is detected in *Daphnia* at the transcript level, but it is absent in *Drosophila*. Abbreviation: Hs, *Homo sapiens*; Dm, *Drosophila melanogaster*; Xl, *Xenopus laevis*; Gg, *Gallus gallus*; Dpul, *Daphnia pulex*. Click here for file

Additional file 9: Figure S7 Phylogenetic tree of the nAChRs. The nAChRs belong to the Cys-loop receptor superfamily. The nAChRs are highly diverse in both vertebrates and invertebrates. *Daphnia* genome encodes 12 putative nAChR genes. Abbreviation: Hs, *Homo sapiens*; Dm, *Drosophila melanogaster*; Gg, *Gallus gallus*; Am, *Apis mellifera*; Dpul, *Daphnia pulex*. Click here for file

Additional file 10: Figure S8 Phylogenetic tree of non-nAChR cys-loop receptors. In addition to the nAChRs, the Cys-loop receptor superfamily also includes 5-hydroxytryptamine type 3 receptors (5-HT3Rs), zinc-activated ion channels (ZACNs), GABA_A_Rs and glycine receptors (GlyRs). The *Daphnia* genome lacks the counterparts of the *Drosophila* genes CG11340, CG6927 and CG7589, which form a separate branch. The pHCl gene seen in *Drosophila* is also absent in *Daphnia*. Click here for file

Additional file 11: Figure S9 Phylogenetic tree of the Deg/ENaC channels. DEG/ENaC channels have been found in nematodes, insects and vertebrates and implicated in a broad spectrum of cellular functions. Mammalian DEG/ENaC channels fall into two major groups (EnaC and ASIC) with 9 members. The invertebrate DEG/ENaC members are highly diverse. Fourteen *Daphnia* ENaC/Deg homologs are detected at transcript level. Click here for file

Additional file 12: Figure S10 Phylogenetic trees of the P2X receptor, ORAI proteins, IP3Rs and RyRs. Two P2X gene transcripts are detected in *Daphnia*, meanwhile, one ORAI, one IP3R and one RyR are detected in *Daphnia*. Click here for file

Additional file 13: Figure S11 Phylogenetic trees of the CLC, CLIC, BEST, tweety and ANO proteins. Chloride channels are a functionally and structurally diverse group of anion selective channels. Chloride channels are poorly understood and classified into ClC, Clic, Best, Tweety and Ano families. There is no sequence homology between these families. Click here for file
