# Supplementary material for: Effects of midazolam, pentobarbital and ketamine on the mRNA expression of ion channels in a model organism Daphnia pulex
Source: BMC Anesthesiol. 2013 Oct 18;13:32. doi: 10.1186/1471-2253-13-32 (PMC3879215; doi:10.1186/1471-2253-13-32)
Supplement: Additional file 1: Table S1 — Gene name, Gene ID, Genome Map, Primer, product size and qPCR efficiency. The forward and reverse primers, gene ID, product size and qPCR efficiency for indicated genes were given, based on the scaffold sequences of the Daphnia Genomics Consortium. All primers were designed using Primer 3 (version 0.4.0). The expression and length of each gene were confirmed by reverse transcription PCR. qPCR efficiency of target genes was calculated by Bio-Rad iQ5 software. [file 1471-2253-13-32-S1.doc]

Table S1 Daphnia ion channel genes and corresponding primers

| **Gene Name** | **Gene Id** | **Genome Map** | **Left Primer** | **Right Primer** | **Product size** | **E%** |
| --- | --- | --- | --- | --- | --- | --- |
| ACHA1 | NCBI_GNO_302064 | scaffold_6:266668-271577 | GGCAACTACGAGGTGACCAT | CCAGGATCCGAATTTCATGT | 156 | 99 |
| ACHA2A | NCBI_GNO_304064 | scaffold_6:301141-327388 | GCTGCCAGACATTCTCCTCT | GTGAGACCACGGACCAAACT | 196 | 98 |
| ACHA2B | NCBI_GNO_116754 | scaffold_135:51886-58620 | TCCTCAACGTCCGCTACCGCAA | TGCTGCTCGATGGACGGAGTGT | 151 | 98 |
| ACHA3 | NCBI_GNO_364544 | scaffold_76:439923-449188 | AGCCCTATTTGGACATCACG | ATCGATATGGCCAAGCTCAC | 160 | 99 |
| ACHA4 | NCBI_GNO_362544 | scaffold_76:418795-438111 | TCAAATTGTCGCAGCTCATC | CGCATTGTTGTAGAGGCTGA | 185 | 104 |
| ACHA5 | NCBI_GNO_318404 | scaffold_48:142222-148562 | TGTTGCGAAGAACCCTATCC | CTCGCCGATAACCATAAGGA | 210 | 97 |
| ACHA6 | NCBI_GNO_502374 | scaffold_42:528124-547599 | GACCTCGTCGAGAAAACGAC | GGAGGGAACGATGAGATTGA | 198 | 101 |
| ACHA7 | NCBI_GNO_500374 | scaffold_42:441617-453635 | CGACAGCACCTATCAAACGA | TAGATCCCATTCGCCGTTAG | 238 | 99 |
| ACHA8 | NCBI_GNO_306064 | scaffold_6:329989-332248 | CGGCAATTACGAGGTGACTT | ACCGAATTTCATGAGGCAAC | 151 | 102 |
| ACHA9 | NCBI_GNO_276693 | scaffold_115:213148-216101 | TTTGGCCCTATTGTGTGGTT | GTCGGTGGCGATACTGATTT | 180 | 102 |
| ACHA10 | NCBI_GNO_1032014 | scaffold_1:3055760-3061059 | TGATGTCCGACCAGTCAAGA | TCATCCGCGTTGTTGTAGAG | 243 | 91 |
| ACHB1 | NCBI_GNO_498374 | scaffold_42:393191-437552 | CATCAGCGTCAACGAGAAAA | TGCACTTGTATCGGACTTCG | 200 | 93 |
| Actin | NCBI_GNO_1270053 | scaffold_5:2220776-2223178 | GGTATGTGCAAGGCTGGATT | GGTGTGGTGCCAGATCTTTT | 225 | 102 |
| ANO1 | NCBI_GNO_736094 | scaffold_9:2000666-2004473 | CTGTTGCATCCTGCTTTTCA | CCGGATAACCGATGAATTTG | 191 | 99 |
| ANO2 | NCBI_GNO_408014 | scaffold_1:549480-554019 | CATCTACCGGATCGTGGTCT | GTTCTGTGCATTTCCCAGGT | 174 | 95 |
| ANO3 | NCBI_GNO_694034 | scaffold_3:1821427-1824360 | TGGCCACGAAACTCACGGAATGG | TGCAACTTGCCACTGAAGCATAGGG | 164 | 99 |
| Best1 | NCBI_GNO_298194 | scaffold_19:1131028-1133548 | GTTGCCACTGTCGCTGCCAGAT | CAGCGATCTTACCAACAGCACACCC | 174 | 103 |
| Best2 | NCBI_GNO_588594 | scaffold_87:271781-273958 | ACATGACGATCCCTTGGCCGGA | TCGGGAAACGACGACGGACTGA | 168 | 95 |
| Best3 | NCBI_GNO_620014 | scaffold_1:1627660-1629372 | GTCGAAGCCGATGTGAATTT | GGAATCGAGACCCAGTCGTA | 179 | 97 |
| Best4 | NCBI_GNO_1176044 | scaffold_4:2999228-3000908 | GTTGCGGCGCTCAATGAAGCTG | GGGCCATCAGGCAAGCGATGAA | 199 | 94 |
| Ca-alpha1D | NCBI_GNO_870024 | scaffold_2:2732330-2746915 | CATCCAAGCTACAACGCTCA | TCCGTCTTGCGTCAACATTA | 207 | 102 |
| Ca-alpha1T | NCBI_GNO_590134 | scaffold_13:1542286-1544831 | ACGCTCACTTTGCCAACTTT | CATCAGGACGAAGATGACGA | 185 | 106 |
| cac | NCBI_GNO_66914 | scaffold_218:112845-133828 | TGTTCCTCTCATCGTCATCG | CTGCTTTGCAGATCCATTCA | 179 | 94 |
| CLC-a1 | NCBI_GNO_414034 | scaffold_3:527692-531608 | CCGACATGTTGGCCACTGCTTGT | CGCCGAAAACCGCCGAGAAGAA | 147 | 105 |
| CLC-a2 | NCBI_GNO_418034 | scaffold_3:537008-543846 | AAGACCGACATGTTGGCCGCTG | GGGCGCTGAAAACCGCTGAGAA | 154 | 102 |
| CLC-a3 | NCBI_GNO_420034 | scaffold_3:544336-549599 | TGGCGAGGCCATGCATCTTTGG | TCACTCCAGCGGAAAAAGCCGC | 116 | 101 |
| CLC-a4 | NCBI_GNO_422034 | scaffold_3:553954-558737 | TCGCCAAAGTTGTGGCCCTCAC | ATTGCACGAGACACCCACAGCG | 197 | 96 |
| CLC-b | NCBI_GNO_38903 | scaffold_210:37002-40879 | GCAGCAGTCGCTGCCGGATTAT | TGGCCGTGGTATGTGCTGAGGA | 281 | 97 |
| CLC-c1 | NCBI_GNO_206284 | scaffold_28:438262-442868 | ACCCGTATGAGTTCCACGAG | GAAGAGCCAGGGCAGTGTAG | 160 | 101 |
| CLC-c2 | NCBI_GNO_338114 | scaffold_11:1198231-1203545 | GATATTTCACGGCGAATGCT | GGCATCATAAATGCCGTCTT | 232 | 104 |
| Clic | NCBI_GNO_772014 | scaffold_1:2076679-2086054 | ACCAACTTTGAAGCCACACC | TTGAGACAAAAGGGCGTTCT | 222 | 99 |
| Clic-like | NCBI_GNO_2066033 | scaffold_3:3615428-3617014 | GTGCAAACTTGTCCGTCTGA | TCAAGGCTGATTTGATGACG | 202 | 108 |
| CNGA1 | NCBI_GNO_852014 | scaffold_1:2360484-2364020 | ACATGGTCTTCCGCAAAGTC | CAAACCTGGTTCGCAATCTT | 203 | 99 |
| CNGA2 | NCBI_GNO_64724 | scaffold_123:97801-110870 | GACAATGGGCGAACTGTTTT | GCGATGGCTTCTAGTCGAAC | 206 | 102 |
| CNGB | NCBI_GNO_362564 | scaffold_80:410366-414133 | AGGATTGTGATCCTGCCTTG | CACCAACAGCCAACAAACTG | 222 | 95 |
| cngl | NCBI_GNO_142404 | scaffold_47:539060-551946 | CCAAGGTGGAGGAGATACCA | TGTCACCAGGCGTGAAAATA | 182 | 101 |
| DEG/ENaC-1 | JGI_V11_94123 | scaffold_1:1595286-1597307 | CGTCACTCCGGCGCTCAACATT | TCGCAGAGTTCGGCCGTGTACT | 168 | 98 |
| DEG/ENaC-2 | NCBI_GNO_300334 | scaffold_33:955732-957968 | ATGCTGATACCTTCGCCCGCCA | CGCCGATATCGCAAAGCAGCGA | 126 | 104 |
| DEG/ENaC-3 | NCBI_GNO_630374 | scaffold_42:1004465-1005994 | GGGCGTCTGCTACTCTTACACGCT | AGAGTGTTGTGCCGTGTGTGCG | 191 | 105 |
| DEG/ENaC-4 | NCBI_GNO_708174 | scaffold_17:1398072-1400458 | ACTTGGGTGAAAACGACGGGCAG | GCGAGTCACGCAAGTCGTGGAT | 148 | 102 |
| DEG/ENaC-5 | NCBI_GNO_413584 | scaffold_9251:433-1220 | GGGATGCCATCATCTGCGCCAA | TTCCGGCCTCCGTAGGCTTTCA | 181 | 102 |
| DEG/ENaC-6 | NCBI_GNO_400244 | scaffold_24:1033873-1036363 | GTTTCCTTGCGAGCCCGGGATTT | AGCTGGCAATACGGTCGGGTGT | 166 | 96 |
| DEG/ENaC-7 | NCBI_GNO_538084 | scaffold_8:1900532-1908312 | CAACACTTCGATGCACGGCCTCA | TCGCCGAGAGCTCGAGTCGAAA | 252 | 94 |
| DEG/ENaC-8 | NCBI_GNO_188894 | scaffold_207:55386-57652 | GAAAATGGAACCACCGGCACCGT | TGAACCCGCGCTCAATCACGTC | 169 | 105 |
| DEG/ENaC-9 | NCBI_GNO_550033 | scaffold_3:1222735-1225206 | ACATTCGTCGGAGTCAAAGG | TGCATTTACATCGCTCTTGC | 184 | 95 |
| DEG/ENaC-10 | NCBI_GNO_532034 | scaffold_3:1208132-1210820 | GCTGCACCAGCTTGCTCAGACA | CTCCGTTGAATGTGCAACACATACCGT | 121 | 98 |
| DEG/ENaC-11 | NCBI_GNO_530034 | scaffold_3:1200971-1206847 | CAAGAAGACTGTAACTGCCGCCCG | GGGCGGCAAGCATTCGCAGATT | 144 | 107 |
| DEG/ENaC-12 | NCBI_GNO_534034 | scaffold_3:1211693-1214358 | TGCAAAACTGCAAACGACACTGCG | ACGGTTCGGAAAGCCCTAGCCA | 200 | 100 |
| DEG/ENaC-13 | NCBI_GNO_536034 | scaffold_3:1215885-1218322 | CAATCGGCCTGTGTCGCCGAAT | TTCCGTTCCGCCGCATTTCCTC | 157 | 101 |
| DEG/ENaC-14 | JGI_V11_98455 | scaffold_9:1330006-1333575 | ACCTGCAACCACCACCAGGACT | GTAGGGCGTCCTCCTCCATACCTGA | 281 | 93 |
| Dpul_8916 | NCBI_GNO_810024 | scaffold_2:2276098-2279994 | GGATTTGGAAGCCGGATACT | GTCCATTGGAAACTCCCTCA | 170 | 98 |
| eag | NCBI_GNO_158434 | scaffold_53:472030-500718 | GACTCGATACGTGCATGGTG | CGTCCTGGATGACCTCTAGC | 249 | 97 |
| erg | NCBI_GNO_402014 | scaffold_1:460048-513231 | TGCGGGTCAAGGAGTTTATC | TTGAGCAAGTGTCGGTTGAG | 181 | 97 |
| GluCl | NCBI_GNO_164584 | scaffold_83:459784-468758 | GCCTGAAAGTGGATCTCCTG | GGCGGAAGTGAGTTGTTGAT | 202 | 107 |
| Glu-RI | NCBI_GNO_152584 | scaffold_83:310215-325671 | CGCCTACATCCTGGTCAGTT | GATTGAGTCCCGATCCTTGA | 176 | 98 |
| Grd | NCBI_GNO_408024 | scaffold_2:536871-548878 | CTAGGTGTCACCACGGTCCT | CCGAAGCCGATTTTAGTGAA | 176 | 97 |
| hclA | NCBI_GNO_254493 | scaffold_65:670723-684475 | TCAGCCTTCCGGAAAATATG | TCATGGAGCACGACAGAGTC | 207 | 109 |
| hclB | NCBI_GNO_18654 | scaffold_102:25033-27646 | GGCCAGGGTTACCTTAGGAG | GGTTCCCATGAAGTGATTGC | 181 | 102 |
| iav | NCBI_GNO_588034 | scaffold_3:1408214-1413760 | CGTAGGGACGTTCTTCTTGC | TGCGTAACCGTACATTTCCA | 241 | 102 |
| Ih | NCBI_GNO_364214 | scaffold_21:652380-711789 | GAGGTCTTCCAACCAGGTGA | AGGACGGCATTGAAGTGTTC | 242 | 94 |
| IP3R | NCBI_GNO_672594 | scaffold_87:507009-516657 | CAAACCGTCGGGTATTAGGA | CGAGGAAGCAAAAGATGAGG | 230 | 103 |
| Ir | NCBI_GNO_1056044 | scaffold_4:2631626-2636109 | TTCAAACGACCATCGGATTT | GGCTTGAGCTCTTTGTTTCG | 161 | 95 |
| Irk2 | NCBI_GNO_1052044 | scaffold_4:2625535-2629364 | CGGGACGATCAACTCTGTTT | CGTGGTCGAAACCAACTTTC | 160 | 103 |
| KaiR1 | NCBI_GNO_96404 | scaffold_47:225688-257255 | TCAGCCGACTCGACTCTTTT | GATTGAGTCCCGATCCTTGA | 242 | 107 |
| KaiR2 | NCBI_GNO_98404 | scaffold_47:276388-288391 | GAAGCCAAGTACGGCATCTC | AGAGCCAAATGTCCAACGTC | 244 | 106 |
| KaiR3 | NCBI_GNO_562594 | scaffold_87:221991-225879 | TCACCCGTGCGATCCCAATCCA | AGAACCACCAAATGCCGGCCAC | 152 | 96 |
| KaiR4 | NCBI_GNO_598014 | scaffold_1:1544842-1549207 | TTCTCGACGGAAAAGCTGAT | GATAACGAAACGGCCAGGTA | 217 | 98 |
| KCNQ | NCBI_GNO_84314 | scaffold_31:219478-225102 | CGTGTTCGTGATGGTCTTTG | CTTCAAACGACCGAGGAATC | 193 | 101 |
| Lcch3 | NCBI_GNO_654024 | scaffold_2:1590779-1594027 | ACCGTCGAAATCGAAAGCTA | CAAGGATAGGCGCTGATAGG | 174 | 106 |
| Nmdar1 | NCBI_GNO_64584 | scaffold_83:38485-50094 | CCATCCTGGAGAAAAAGCAA | GAATCGACCGAAAGGTGAAA | 152 | 93 |
| Nmdar2 | NCBI_GNO_356034 | scaffold_3:254236-273561 | ATGCACTACCACATGCGAAA | TAACCTGTCATGGCGTACCA | 176 | 103 |
| Nmdar3 | NCBI_GNO_498134 | scaffold_13:1301554-1306777 | GATCTGTGGATCGGCATCTT | CCCAGACGTTGATGAGGAAC | 223 | 104 |
| NACH | NCBI_GNO_478454 | scaffold_58:716468-723956 | TTCCCAGAGGCTTTCATGTC | AGCTGAGCACGATCAGTGTG | 160 | 97 |
| NaCP60E | NCBI_GNO_632024 | scaffold_2:1478261-1509262 | GTACGTGTTCCTGGGCATCT | GGATGATGGAAACGGTTTTG | 218 | 105 |
| nompC | NCBI_GNO_76604 | scaffold_88:266953-274618 | GCGGCCCTTAAAGGCTATAC | CGTTTCGGCTCCTAATTCAA | 162 | 99 |
| nan | NCBI_GNO_480074 | scaffold_7:1739234-1743523 | ACGCATTATCGGGAAGTTTG | TGAAGTACGTTCTCGCCGTA | 188 | 106 |
| ORAI | NCBI_GNO_352053 | scaffold_5:840373-842075 | GCAATTTTGGTTCCATTTGTG | TTCCCGAGTGGTCAACTCAT | 159 | 97 |
| Ork1 | NCBI_GNO_414084 | scaffold_8:1234386-1238283 | TGTTGCCGTTACTGTTGCAT | TGATCTCCTAACGCAGCAAA | 138 | 94 |
| pain | NCBI_GNO_366134 | scaffold_13:848808-852942 | GTCGACATTGTCCGTCCTTT | TCGTCCAATCGGAATGTTCT | 205 | 103 |
| para | NCBI_GNO_386214 | scaffold_21:809175-839828 | TCATTGCCTTAGCGTACGTG | CGAGAACAGCGTCAAGATGA | 182 | 101 |
| pyx1 | NCBI_GNO_934014 | scaffold_1:2645974-2649331 | CCCGACGCCTTGACAGCCATTT | TTGCTGGTTGCCCCCTGGTACT | 129 | 105 |
| Pyx2 | NCBI_GNO_114614 | scaffold_90:260184-263112 | TACCACCGTTGCATGGAATA | AATCCATTTCGCCTGTCATC | 164 | 97 |
| P2XL1 | NCBI_GNO_488034 | scaffold_3:863221-865354 | AGAGATCCCTTCTGCCCTGT | GGAGCGATGTTGGTATTTGG | 200 | 103 |
| P2XL2 | NCBI_GNO_490034 | scaffold_3:867144-868791 | AGAGATCCCTTCTGCCCTGT | GGAGCGATGTTGGTATTTGG | 200 | 100 |
| Rdl | NCBI_GNO_104424 | scaffold_51:132163-203101 | ACGACGTCCAACGAGTTTCT | CCCAGCCAGTGGTAAGTGAT | 182 | 99 |
| RdlL | NCBI_GNO_144104 | scaffold_10:35172-43801 | GTCGCCAAGTCGCACTGCAAGA | GCGAGGCGCCATACATGACGTT | 133 | 105 |
| RYR | NCBI_GNO_1700023 | scaffold_2:2916754-2942066 | GTGGCTCTCAGGCTTTTGAC | CCATCTTGGAAAGGGAATCA | 154 | 105 |
| Shab | NCBI_GNO_304244 | scaffold_24:809440-819647 | GTGGAACTGTGCGACGACTA | TCCAGGTAGAGCTCGTCGAT | 182 | 108 |
| Shal | NCBI_GNO_402354 | scaffold_38:133785-168797 | TTGTGACGTTGCGAGTCTTC | CAGTGGCGAAGATGATGATG | 156 | 102 |
| Shaker | NCBI_GNO_294444 | scaffold_56:235328-247670 | CGCGTCGTTATCAATGTGAG | CTCCGCAATCTACCACCACT | 200 | 95 |
| Shaw | NCBI_GNO_52544 | scaffold_75:24735-35405 | GCATCATGAGGCTGTTCAAA | GGGATGCTGTTGAAGTCGTT | 193 | 92 |
| Shawl1 | NCBI_GNO_588084 | scaffold_8:2040111-2051826 | CTACTACAGGACGGGCAAGC | TCGGTTGGCTTTTCTGTTTC | 195 | 100 |
| Shawl2 | NCBI_GNO_496114 | scaffold_11:1758737-1773486 | CCAACTTGCGAGTGCCCGTCAT | AGAAGGCTTGATGGGGCTCGGT | 183 | 101 |
| slo | NCBI_GNO_758094 | scaffold_9:2127503-2157277 | GACGGGAATGTTTCACTGGT | TGCTTCAGCTCGTGGTAATG | 195 | 96 |
| slo-like | NCBI_GNO_254664 | scaffold_107:205671-218930 | CGTCCAACATGCGTTTTATG | CAACATGCTGGCTGAGAAAA | 155 | 102 |
| SK | NCBI_GNO_1056014 | scaffold_1:3154720-3181507 | CCGAGTCATGCTTCTTCACA | CACAGCGACACCATGAAGAC | 156 | 103 |
| Task6 | NCBI_GNO_244154 | scaffold_15:371433-374937 | GCCCTGGAATCTGATAACGA | CATTGTTTGCCAGCGTAATG | 152 | 104 |
| Task7 | NCBI_GNO_194124 | scaffold_12:442452-443393 | TACTCATCCGCAACGTCAAA | TAACCAGGGCGATTGGTAAG | 247 | 102 |
| TRESK | NCBI_GNO_516144 | scaffold_14:1247127-1251701 | ACCCACGAGCTCAACGTCCTGT | CGGCGCAATGTTGCCATAACCG | 189 | 93 |
| TRP | NCBI_GNO_298094 | scaffold_9:569231-574806 | CTTCCCATTGTTCCCTCTGA | GGATCCATACCCGGACCTAT | 211 | 102 |
| TRPA5 | NCBI_GNO_266234 | scaffold_19:789915-790232 | TGAAAGTGTGGAAACGCTGA | CGTTCCACCTTCATTGTCCT | 169 | 98 |
| trpgamma | NCBI_GNO_300444 | scaffold_56:282882-311121 | CGAGCTTTGGCTAGTCCATC | TGTTCGAAAGCAGGACCTTT | 239 | 96 |
| TRPL | NCBI_GNO_22374 | scaffold_41:25936-35110 | GCCAGTTTCGGTTTGGTAGA | CGAGCGAATTTCCATTCTGT | 197 | 100 |
| TRPM1 | NCBI_GNO_256254 | scaffold_25:507501-512608 | CTTGGAACCGAGTCGACATT | AGAAATCTGGCCATTTGCAC | 160 | 99 |
| TRPM2 | NCBI_GNO_466254 | scaffold_25:1074184-1080408 | TCGACATTGCCCGCTCCGAGAT | GCCGGCCCTTGTTTCGAGTTGT | 196 | 101 |
| TRPML | NCBI_GNO_214394 | scaffold_45:599135-602228 | ACAGCAACGAGCACAAACAG | TCGCGAAAAGATGCAAATAA | 233 | 100 |
| tty | NCBI_GNO_242114 | scaffold_11:707862-711016 | GGTTCTCGGTGTGACGGCGTTT | AGGCGATCCGCTAGTGGGAAACT | 198 | 100 |
| TPC | NCBI_GNO_324294 | scaffold_29:938802-942548 | CAGTGGAGCCGCATTTATTT | GCAGATTCTACCGCAAGGAG | 203 | 97 |
| TWIK | NCBI_GNO_40753 | scaffold_134:195768-201118 | GCAAATGCGTCAACTTACGA | GGGTTGTGTCTTCGGGACTA | 229 | 106 |

I
