# Supplementary material for: Effects of midazolam, pentobarbital and ketamine on the mRNA expression of ion channels in a model organism Daphnia pulex
Source: BMC Anesthesiol. 2013 Oct 18;13:32. doi: 10.1186/1471-2253-13-32 (PMC3879215; doi:10.1186/1471-2253-13-32)
Supplement: Additional file 2: Table S2 — Gene name, synonyms of the gene name and Uniprot accession numbers used in phylogenies. [file 1471-2253-13-32-S2.doc]

| **Figure** | **Gene Name** | **Synonyms** | **Database number** | **Species** |
| --- | --- | --- | --- | --- |
| **Fig S1** | Ce_irk-1 |  | P52192 | Caenorhabditis elegans |
|  | Ce_irk-2 |  | H2KYE6 | Caenorhabditis elegans |
|  | Ce_irk-3 |  | Q93849 | Caenorhabditis elegans |
|  | Dm_Ir |  | Q95UP7 | Drosophila melanogaster |
|  | Dm_Irk2 |  | Q9VCL9 | Drosophila melanogaster |
|  | Dm_Irk3 |  | Q9VJ56 | Drosophila melanogaster |
|  | Dpul_Ir |  | NCBI_GNO_1056044 | Daphnia pulex |
|  | Dpul_Irk2 |  | NCBI_GNO_1052044 | Daphnia pulex |
|  | Hs_KCNJ1_Kir1.1 |  | P48048 | Homo sapiens |
|  | Hs_KCNJ2_Kir2.1 | IRK1 | P63252 | Homo sapiens |
|  | Hs_KCNJ3_Kir3.1 | GIRK1 | P48549 | Homo sapiens |
|  | Hs_KCNJ4_Kir2.3 | IRK3 | P48050 | Homo sapiens |
|  | Hs_KCNJ5_Kir3.4 | GIRK4 | P48544 | Homo sapiens |
|  | Hs_KCNJ6_Kir3.2 | GIRK2 | P48051 | Homo sapiens |
|  | Hs_KCNJ8_Kir6.1 |  | Q15842 | Homo sapiens |
|  | Hs_KCNJ9_Kir3.3 | GIRK3 | Q92806 | Homo sapiens |
|  | Hs_KCNJ10_Kir1.2 |  | P78508 | Homo sapiens |
|  | Hs_KCNJ11_Kir6.2 |  | D2K1F9 | Homo sapiens |
|  | Hs_KCNJ12_Kir2.2 | IRK2 | Q14500 | Homo sapiens |
|  | Hs_KCNJ13_Kir7.1 |  | O60928 | Homo sapiens |
|  | Hs_KCNJ14_Kir2.4 | IRK4 | Q9UNX9 | Homo sapiens |
|  | Hs_KCNJ15_Kir1.3 |  | Q99712 | Homo sapiens |
|  | Hs_KCNJ16_Kir5.1 |  | Q9NPI9 | Homo sapiens |
|  |  |  |  |  |
|  |  |  |  |  |
| **Fig S2** | Dm_CG17922 |  | Q9W2D5 | Drosophila melanogaster |
|  | Dm_CG34363 |  | A8DY94 | Drosophila melanogaster |
|  | Dm_CG42260 |  | Q9W201 | Drosophila melanogaster |
|  | Dm_Cng |  | Q24278 | Drosophila melanogaster |
|  | Dm_cngl |  | Q9U5E2 | Drosophila melanogaster |
|  | Dm_eag |  | Q02280 | Drosophila melanogaster |
|  | Dm_elk |  | Q23974 | Drosophila melanogaster |
|  | Dm_erg |  | Q7JPB9 | Drosophila melanogaster |
|  | Dm_Ih |  | B7YZE8 | Drosophila melanogaster |
|  | Dm_KCNQ |  | B7YZR4 | Drosophila melanogaster |
|  | Dm_Shab |  | P17970 | Drosophila melanogaster |
|  | Dm_Shaker |  | P08510 | Drosophila melanogaster |
|  | Dm_Shal |  | P17971 | Drosophila melanogaster |
|  | Dm_Shaw |  | P17972 | Drosophila melanogaster |
|  | Dm_Shawl |  | A8DYR5 | Drosophila melanogaster |
|  | Dm_SK |  | Q7KVW5 | Drosophila melanogaster |
|  | Dm_slo |  | Q03720 | Drosophila melanogaster |
|  | Dpul_CNGA1 |  | NCBI_GNO_852014 | Daphnia pulex |
|  | Dpul_CNGA2 |  | NCBI_GNO_64724 | Daphnia pulex |
|  | Dpul_CNGB |  | NCBI_GNO_362564 | Daphnia pulex |
|  | Dpul_cngl |  | NCBI_GNO_142404 | Daphnia pulex |
|  | Dpul_eag |  | NCBI_GNO_158434 | Daphnia pulex |
|  | Dpul_erg |  | NCBI_GNO_402014 | Daphnia pulex |
|  | Dpul_Ih |  | NCBI_GNO_364214 | Daphnia pulex |
|  | Dpul_KCNQ |  | NCBI_GNO_84314 | Daphnia pulex |
|  | Dpul_Shab |  | NCBI_GNO_304244 | Daphnia pulex |
|  | Dpul_Shal |  | NCBI_GNO_402354 | Daphnia pulex |
|  | Dpul_Shaker |  | NCBI_GNO_294444 | Daphnia pulex |
|  | Dpul_Shaw |  | NCBI_GNO_52544 | Daphnia pulex |
|  | Dpul_Shawl1 |  | NCBI_GNO_588084 | Daphnia pulex |
|  | Dpul_Shawl2 |  | NCBI_GNO_496114 | Daphnia pulex |
|  | Dpul_slo |  | NCBI_GNO_758094 | Daphnia pulex |
|  | Dpul_slo-like |  | NCBI_GNO_254664 | Daphnia pulex |
|  | Dpul_SK |  | NCBI_GNO_1056014 | Daphnia pulex |
|  | Hs_CNGA1 | CNG1 | P29973 | Homo sapiens |
|  | Hs_CNGA2 | CNG2 | Q16280 | Homo sapiens |
|  | Hs_CNGA3 | CNG3 | Q16281 | Homo sapiens |
|  | Hs_CNGA4 | CNG4 | Q8IV77 | Homo sapiens |
|  | Hs_CNGB1 | CNG5 | Q14028 | Homo sapiens |
|  | Hs_CNGB3 | CNG6 | Q9NQW8 | Homo sapiens |
|  | Hs_HCN1 | BCNG1 | O60741 | Homo sapiens |
|  | Hs_HCN2 | BCNG2 | Q9UL51 | Homo sapiens |
|  | Hs_HCN3 |  | Q9P1Z3 | Homo sapiens |
|  | Hs_HCN4 |  | Q9Y3Q4 | Homo sapiens |
|  | Hs_KCNA1_Kv1.1 |  | Q09470 | Homo sapiens |
|  | Hs_KCNA2_Kv1.2 |  | P16389 | Homo sapiens |
|  | Hs_KCNA3_Kv1.3 |  | P22001 | Homo sapiens |
|  | Hs_KCNA4_Kv1.4 |  | P22459 | Homo sapiens |
|  | Hs_KCNA5_Kv1.5 |  | P22460 | Homo sapiens |
|  | Hs_KCNB1_Kv2.1 |  | Q14721 | Homo sapiens |
|  | Hs_KCNB2_Kv2.2 |  | Q92953 | Homo sapiens |
|  | Hs_KCNC1_Kv3.1 |  | P48547 | Homo sapiens |
|  | Hs_KCNC2_Kv3.2 |  | Q96PR1 | Homo sapiens |
|  | Hs_KCNC3_Kv3.3 |  | Q14003 | Homo sapiens |
|  | Hs_KCNC4_Kv3.4 |  | Q03721 | Homo sapiens |
|  | Hs_KCND1_Kv4.1 |  | Q9NSA2 | Homo sapiens |
|  | Hs_KCND2_Kv4.2 |  | Q9NZV8 | Homo sapiens |
|  | Hs_KCND3_Kv4.3 |  | Q9UK17 | Homo sapiens |
|  | Hs_KCNH1_Kv10.1 | EAG1 | O95259 | Homo sapiens |
|  | Hs_KCNH2_Kv11.1 | ERG1 | Q12809 | Homo sapiens |
|  | Hs_KCNH3_Kv12.2 |  | Q9ULD8 | Homo sapiens |
|  | Hs_KCNH4_Kv12.3 |  | Q9UQ05 | Homo sapiens |
|  | Hs_KCNH5_Kv10.2 | EAG2 | Q8NCM2 | Homo sapiens |
|  | Hs_KCNH6_Kv11.2 | ERG2 | Q9H252 | Homo sapiens |
|  | Hs_KCNH7_Kv11.3 | ERG3 | Q9NS40 | Homo sapiens |
|  | Hs_KCNH8_Kv12.1 |  | Q96L42 | Homo sapiens |
|  | Hs_KCNMA_KCa1.1 | SLO | Q12791 | Homo sapiens |
|  | Hs_KCNN1_KCa2.1 | SK | Q92952 | Homo sapiens |
|  | Hs_KCNN2_KCa2.2 |  | Q9H2S1 | Homo sapiens |
|  | Hs_KCNN3_KCa2.3 |  | Q9UGI6 | Homo sapiens |
|  | Hs_KCNN4_KCa3.1 |  | O15554 | Homo sapiens |
|  | Hs_KCNT1_KCa4.1 |  | Q5JUK3 | Homo sapiens |
|  | Hs_KCNT2_Slo2.1 | SLICK | Q6UVM3 | Homo sapiens |
|  | Hs_KCNQ1_Kv7.1 |  | P51787 | Homo sapiens |
|  | Hs_KCNQ2_Kv7.2 |  | O43526 | Homo sapiens |
|  | Hs_KCNQ3_Kv7.3 |  | O43525 | Homo sapiens |
|  | Hs_KCNQ4_Kv7.4 |  | P56696 | Homo sapiens |
|  | Hs_KCNQ5_Kv7.5 |  | Q9NR82 | Homo sapiens |
|  |  |  |  |  |
|  |  |  |  |  |
| **Fig S3** | Ce_twk-46 |  | Q9NEV3 | Caenorhabditis elegans |
|  | Dm_CG10864 |  | Q9VE68 | Drosophila melanogaster |
|  | Dm_Ork1 |  | Q94526 | Drosophila melanogaster |
|  | Dm_Task6 |  | Q9VFS9 | Drosophila melanogaster |
|  | Dm_Task7 |  | Q3ZZY0 | Drosophila melanogaster |
|  | Dpul_Ork1 |  | NCBI_GNO_414084 | Daphnia pulex |
|  | Dpul_Task6 |  | NCBI_GNO_244154 | Daphnia pulex |
|  | Dpul_Task7 |  | NCBI_GNO_194124 | Daphnia pulex |
|  | Dpul_TRESK |  | NCBI_GNO_516144 | Daphnia pulex |
|  | Dpul_TWIK |  | NCBI_GNO_40753 | Daphnia pulex |
|  | Hs_KCNK1_K2P1.1 | TWIK1 | O00180 | Homo sapiens |
|  | Hs_KCNK2_K2P2.1 | TREK1 | O95069 | Homo sapiens |
|  | Hs_KCNK3_K2P3.1 | TASK1 | O14649 | Homo sapiens |
|  | Hs_KCNK4_K2P4.1 | TRAAK | Q9NYG8 | Homo sapiens |
|  | Hs_KCNK5_K2P5.1 | TASK2 | O95279 | Homo sapiens |
|  | Hs_KCNK6_K2P6.1 | TWIK2 | Q9Y257 | Homo sapiens |
|  | Hs_KCNK7_K2P7.1 |  | Q9Y2U2 | Homo sapiens |
|  | Hs_KCNK9_K2P9.1 | TASK3 | Q9NPC2 | Homo sapiens |
|  | Hs_KCNK10_K2P10.1 | TREK2 | P57789 | Homo sapiens |
|  | Hs_KCNK12_K2P12.1 | THIK2 | Q9HB15 | Homo sapiens |
|  | Hs_KCNK13_K2P13.1 | THIK1 | Q9HB14 | Homo sapiens |
|  | Hs_KCNK15_K2P15.1 | TASK5 | Q9H427 | Homo sapiens |
|  | Hs_KCNK16_K2P16.1 | TALK1 | Q96T55 | Homo sapiens |
|  | Hs_KCNK17_K2P17.1 | TASK4 | Q96T54 | Homo sapiens |
|  | Hs_KCNK18_K2P18.1 | TRESK | Q7Z418 | Homo sapiens |
|  |  |  |  |  |
|  |  |  |  |  |
| **Fig S4** | Am_TPC |  | H9KF69 | Apis mellifera |
|  | Dm_Ca-alpha1D |  | Q24270 | Drosophila melanogaster |
|  | Dm_Ca-alpha1T |  | Q9W433 | Drosophila melanogaster |
|  | Dm_cac |  | P91645 | Drosophila melanogaster |
|  | Dm_NACH |  | A8JUW5 | Drosophila melanogaster |
|  | Dm_NaCP60E |  | Q9W0Y8 | Drosophila melanogaster |
|  | Dm_para |  | P35500 | Drosophila melanogaster |
|  | Dpul_Ca-alpha1D |  | NCBI_GNO_870024 | Daphnia pulex |
|  | Dpul_Ca-alpha1T |  | NCBI_GNO_590134 | Daphnia pulex |
|  | Dpul_cac |  | NCBI_GNO_66914 | Daphnia pulex |
|  | Dpul_NACH |  | NCBI_GNO_478454 | Daphnia pulex |
|  | Dpul_NaCP60E |  | NCBI_GNO_632024 | Daphnia pulex |
|  | Dpul_para |  | NCBI_GNO_386214 | Daphnia pulex |
|  | Dpul_TPC |  | NCBI_GNO_324294 | Daphnia pulex |
|  | Hs_CACNA1A_Cav2.1 |  | O00555 | Homo sapiens |
|  | Hs_CACNA1B_Cav2.2 |  | Q00975 | Homo sapiens |
|  | Hs_CACNA1C_Cav1.2 |  | Q13936 | Homo sapiens |
|  | Hs_CACNA1D_Cav1.3 |  | Q01668 | Homo sapiens |
|  | Hs_CACNA1E_Cav2.3 |  | Q15878 | Homo sapiens |
|  | Hs_CACNA1F_Cav1.4 |  | O60840 | Homo sapiens |
|  | Hs_CACNA1G_Cav3.1 |  | O43497 | Homo sapiens |
|  | Hs_CACNA1H_Cav3.2 |  | O95180 | Homo sapiens |
|  | Hs_CACNA1I_Cav3.3 |  | Q9P0X4 | Homo sapiens |
|  | Hs_CACNA1S_Cav1.1 |  | Q13698 | Homo sapiens |
|  | Hs_NALCN |  | Q8IZF0 | Homo sapiens |
|  | Hs_SCN1A _Nav1.1 |  | P35498 | Homo sapiens |
|  | Hs_SCN2A_Nav1.2 |  | Q99250 | Homo sapiens |
|  | Hs_SCN3A_Nav1.3 |  | Q9NY46 | Homo sapiens |
|  | Hs_SCN4A_Nav1.4 |  | P35499 | Homo sapiens |
|  | Hs_SCN5A_Nav1.5 |  | Q14524 | Homo sapiens |
|  | Hs_SCN7A |  | Q01118 | Homo sapiens |
|  | Hs_SCN8A_Nav1.6 |  | Q9UQD0 | Homo sapiens |
|  | Hs_SCN9A_Nav1.7 |  | Q15858 | Homo sapiens |
|  | Hs_SCN10A_Nav1.8 |  | Q9Y5Y9 | Homo sapiens |
|  | Hs_SCN11A_Nav1.9 |  | Q9UI33 | Homo sapiens |
|  | Hs_TPCN1 |  | Q9ULQ1 | Homo sapiens |
|  | Hs_TPCN2 |  | Q8NHX9 | Homo sapiens |
|  |  |  |  |  |
|  |  |  |  |  |
| **Fig S5** | Am_Wtrw |  | H9KBP4 | Apis mellifera |
|  | Am_iav |  | H9K7C0 | Apis mellifera |
|  | Am_nan |  | H9KQR3 | Apis mellifera |
|  | Am_nompC |  | H9JYZ3 | Apis mellifera |
|  | Am_pain |  | H9JYZ1 | Apis mellifera |
|  | Am_pyx |  | H9KA86 | Apis mellifera |
|  | Am_HsTRPA |  | H9KH51 | Apis mellifera |
|  | Am_TRPA5 |  | H9KA86 | Apis mellifera |
|  | Ce_PKD1 |  | Q09624 | Caenorhabditis elegans |
|  | Ce_PKD2 |  | Q9U1S7 | Caenorhabditis elegans |
|  | Ce_trpa-1 |  | Q18297 | Caenorhabditis elegans |
|  | Dm_AMO |  | Q6T3S5 | Drosophila melanogaster |
|  | Dm_nan |  | Q9VUD5 | Drosophila melanogaster |
|  | Dm_iav |  | Q9W3W0 | Drosophila melanogaster |
|  | Dm_nompC |  | Q9VMR4 | Drosophila melanogaster |
|  | Dm_pyx |  | Q9W0T5 | Drosophila melanogaster |
|  | Dm_pain |  | Q9W0Y6 | Drosophila melanogaster |
|  | Dm_TRPA1 |  | Q7Z020 | Drosophila melanogaster |
|  | Dm_trpgamma |  | Q9VJJ7 | Drosophila melanogaster |
|  | Dm_TRPM |  | A8DYE2 | Drosophila melanogaster |
|  | Dm_TRPML |  | Q9VW35 | Drosophila melanogaster |
|  | Dm_TRP |  | P19334 | Drosophila melanogaster |
|  | Dm_TRPL |  | P48994 | Drosophila melanogaster |
|  | Dm_wtrw |  | Q9VHY7 | Drosophila melanogaster |
|  | Dpul_iav |  | NCBI_GNO_588034 | Daphnia pulex |
|  | Dpul_nan |  | NCBI_GNO_480074 | Daphnia pulex |
|  | Dpul_nompC |  | NCBI_GNO_76604 | Daphnia pulex |
|  | Dpul_pain |  | NCBI_GNO_366134 | Daphnia pulex |
|  | Dpul_pyx1 |  | NCBI_GNO_934014 | Daphnia pulex |
|  | Dpul_Pyx2 |  | NCBI_GNO_114614 | Daphnia pulex |
|  | Dpul_trpgamma |  | NCBI_GNO_300444 | Daphnia pulex |
|  | Dpul_TRP |  | NCBI_GNO_298094 | Daphnia pulex |
|  | Dpul_TRPA5 |  | NCBI_GNO_266234 | Daphnia pulex |
|  | Dpul_TRPL |  | NCBI_GNO_22374 | Daphnia pulex |
|  | Dpul_TRPM1 |  | NCBI_GNO_256254 | Daphnia pulex |
|  | Dpul_TRPM2 |  | NCBI_GNO_466254 | Daphnia pulex |
|  | Dpul_TRPML |  | NCBI_GNO_214394 | Daphnia pulex |
|  | Dr_nompc |  | Q7T1G6 | Danio rerio |
|  | Hs_TRPA1 |  | O75762 | Homo sapiens |
|  | Hs_TRPC1 |  | P48995 | Homo sapiens |
|  | Hs_TRPC3 |  | Q13507 | Homo sapiens |
|  | Hs_TRPC4 |  | Q9UBN4 | Homo sapiens |
|  | Hs_TRPC5 |  | Q9UL62 | Homo sapiens |
|  | Hs_TRPC6 |  | Q9Y210 | Homo sapiens |
|  | Hs_TRPC7 |  | Q9HCX4 | Homo sapiens |
|  | Hs_TRPM1 |  | Q7Z4N2 | Homo sapiens |
|  | Hs_TRPM2 |  | O94759 | Homo sapiens |
|  | Hs_TRPM3 |  | Q9HCF6 | Homo sapiens |
|  | Hs_TRPM4 |  | Q8TD43 | Homo sapiens |
|  | Hs_TRPM5 |  | Q9NZQ8 | Homo sapiens |
|  | Hs_TRPM6 |  | Q9BX84 | Homo sapiens |
|  | Hs_TRPM7 |  | Q96QT4 | Homo sapiens |
|  | Hs_TRPM8 |  | Q7Z2W7 | Homo sapiens |
|  | Hs_TRPML1 |  | Q9GZU1 | Homo sapiens |
|  | Hs_TRPML2 |  | Q8IZK6 | Homo sapiens |
|  | Hs_TRPML3 |  | Q8TDD5 | Homo sapiens |
|  | Hs_TRPV1 |  | Q8NER1 | Homo sapiens |
|  | Hs_TRPV2 |  | Q9Y5S1 | Homo sapiens |
|  | Hs_TRPV3 |  | Q8NET8 | Homo sapiens |
|  | Hs_TRPV4 |  | Q9HBA0 | Homo sapiens |
|  | Hs_TRPV5 |  | Q9NQA5 | Homo sapiens |
|  | Hs_TRPV6 |  | Q9H1D0 | Homo sapiens |
|  | Hs_PKD1 |  | P98161 | Homo sapiens |
|  | Hs_PKD2 |  | Q13563 | Homo sapiens |
|  |  |  |  |  |
|  |  |  |  |  |
| **Fig S6** | Dm_CG11155 |  | Q9V4A0 | Drosophila melanogaster |
|  | Dm_CG3822 |  | Q9VDH5 | Drosophila melanogaster |
|  | Dm_CG5621 |  | Q9VDH2 | Drosophila melanogaster |
|  | Dm_CG9935 |  | B6IDJ4 | Drosophila melanogaster |
|  | Dm_clumsy |  | Q9VIE2 | Drosophila melanogaster |
|  | Dm_Glu-RI |  | Q03445 | Drosophila melanogaster |
|  | Dm_Glu-RIB |  | Q9TVG7 | Drosophila melanogaster |
|  | Dm_GluRIIA |  | Q9VMP4 | Drosophila melanogaster |
|  | Dm_GluRIIB |  | Q9VMP3 | Drosophila melanogaster |
|  | Dm_GluRIIC |  | Q9VPV3 | Drosophila melanogaster |
|  | Dm_GluRIID |  | Q9TVI0 | Drosophila melanogaster |
|  | Dm_GluRIIE |  | Q0KI42 | Drosophila melanogaster |
|  | Dm_Nmdar1 |  | Q24418 | Drosophila melanogaster |
|  | Dm_Nmdar2 |  | Q8MM14 | Drosophila melanogaster |
|  | Dpul_Glu-RI |  | NCBI_GNO_152584 | Daphnia pulex |
|  | Dpul_KaiR1 |  | NCBI_GNO_96404 | Daphnia pulex |
|  | Dpul_KaiR2 |  | NCBI_GNO_98404 | Daphnia pulex |
|  | Dpul_KaiR3 |  | NCBI_GNO_562594 | Daphnia pulex |
|  | Dpul_KaiR4 |  | NCBI_GNO_598014 | Daphnia pulex |
|  | Dpul_Nmdar1 |  | NCBI_GNO_64584 | Daphnia pulex |
|  | Dpul_Nmdar2 |  | NCBI_GNO_356034 | Daphnia pulex |
|  | Dpul_Nmdar3 |  | NCBI_GNO_498134 | Daphnia pulex |
|  | Gg_KBP |  | P19439 | Gallus gallus |
|  | Hs_GRIA1 | GLUR1 | P42261 | Homo sapiens |
|  | Hs_GRIA2 | GLUR2 | P42262 | Homo sapiens |
|  | Hs_GRIA3 | GLUR3 | P42263 | Homo sapiens |
|  | Hs_GRIA4 | GLUR4 | P48058 | Homo sapiens |
|  | Hs_GRID1 |  | Q9ULK0 | Homo sapiens |
|  | Hs_GRID2 |  | O43424 | Homo sapiens |
|  | Hs_GRIK1 | GLUR5 | P39086 | Homo sapiens |
|  | Hs_GRIK2 | GLUR6 | Q13002 | Homo sapiens |
|  | Hs_GRIK3 | GlUR7 | Q13003 | Homo sapiens |
|  | Hs_GRIK4 |  | Q16099 | Homo sapiens |
|  | Hs_GRIK5 |  | Q16478 | Homo sapiens |
|  | Hs_GRIN1 | NMDAR1 | Q05586 | Homo sapiens |
|  | Hs_GRIN2A | NMDAR2A | Q12879 | Homo sapiens |
|  | Hs_GRIN2B | NMDAR2B | Q13224 | Homo sapiens |
|  | Hs_GRIN2C | NMDAR2C | Q14957 | Homo sapiens |
|  | Hs_GRIN2D | NMDAR2D | O15399 | Homo sapiens |
|  | Hs_GRIN3A | NMDAR3A | Q8TCU5 | Homo sapiens |
|  | Hs_GRIN3B | NMDAR3B | O60391 | Homo sapiens |
|  | Xl_kbp |  | Q91756 | Xenopus laevis |
|  |  |  |  |  |
|  |  |  |  |  |
| **Fig S7** | Am_ACHA1 |  | A0EIZ1 | Apis mellifera |
|  | Am_ACHA2 | nAChRa2 | Q6QHT2 | Apis mellifera |
|  | Am_ACHA3 |  | A0EIZ2 | Apis mellifera |
|  | Am_ACHA4 |  | A0EIZ3 | Apis mellifera |
|  | Am_ACHA5 |  | Q6Q069 | Apis mellifera |
|  | Am_ACHA6 |  | A0EIZ5 | Apis mellifera |
|  | Am_ACHA7 |  | Q6RFT1 | Apis mellifera |
|  | Am_ACHA8 |  | Q8MUR0 | Apis mellifera |
|  | Am_ACHA9 |  | A0EIZ7 | Apis mellifera |
|  | Am_ACHB1 |  | A0EIZ8 | Apis mellifera |
|  | Am_ACHB2 |  | A0EIZ9 | Apis mellifera |
|  | Dm_ACHA1 | nAcRalpha-96Aa | P09478 | Drosophila melanogaster |
|  | Dm_ACHA2 | nAcRalpha-96Ab | P17644 | Drosophila melanogaster |
|  | Dm_ACHA3 | nAcRalpha-7E | O18394 | Drosophila melanogaster |
|  | Dm_ACHA4 | nAcRalpha-80B | Q9NFR5 | Drosophila melanogaster |
|  | Dm_ACHA5 | nAcRalpha-34E | Q7KT97 | Drosophila melanogaster |
|  | Dm_ACHA6 | nAcRalpha-30D | Q9VL79 | Drosophila melanogaster |
|  | Dm_ACHA7 | nAcR-alpha-18C | Q9VWI9 | Drosophila melanogaster |
|  | Dm_ACHB1 | nAcRbeta-64B | P04755 | Drosophila melanogaster |
|  | Dm_ACHB2 | nAcRbeta-96A | P25162 | Drosophila melanogaster |
|  | Dm_ACHB3 | nAcRbeta-21C | Q9VPQ8 | Drosophila melanogaster |
|  | Dpul_ACHA1 |  | NCBI_GNO_302064 | Daphnia pulex |
|  | Dpul_ACHA2A |  | NCBI_GNO_304064 | Daphnia pulex |
|  | Dpul_ACHA2B |  | NCBI_GNO_116754 | Daphnia pulex |
|  | Dpul_ACHA3 |  | NCBI_GNO_364544 | Daphnia pulex |
|  | Dpul_ACHA4 |  | NCBI_GNO_362544 | Daphnia pulex |
|  | Dpul_ACHA5 |  | NCBI_GNO_318404 | Daphnia pulex |
|  | Dpul_ACHA6 |  | NCBI_GNO_502374 | Daphnia pulex |
|  | Dpul_ACHA7 |  | NCBI_GNO_500374 | Daphnia pulex |
|  | Dpul_ACHA8 |  | NCBI_GNO_306064 | Daphnia pulex |
|  | Dpul_ACHA9 |  | NCBI_GNO_276693 | Daphnia pulex |
|  | Dpul_ACHA10 |  | NCBI_GNO_1032014 | Daphnia pulex |
|  | Dpul_ACHB1 |  | NCBI_GNO_498374 | Daphnia pulex |
|  | Gg_ACHA8 |  | Q03481 | Gallus gallus |
|  | Hs_CHRNA1 |  | P02708 | Homo sapiens |
|  | Hs_CHRNA2 |  | Q15822 | Homo sapiens |
|  | Hs_CHRNA3 |  | P32297 | Homo sapiens |
|  | Hs_CHRNA4 |  | P43681 | Homo sapiens |
|  | Hs_CHRNA5 |  | P30532 | Homo sapiens |
|  | Hs_CHRNA6 |  | Q15825 | Homo sapiens |
|  | Hs_CHRNA7 |  | P36544 | Homo sapiens |
|  | Hs_CHRNA9 |  | Q9UGM1 | Homo sapiens |
|  | Hs_CHRNA10 |  | Q9GZZ6 | Homo sapiens |
|  | Hs_CHRNB1 |  | P11230 | Homo sapiens |
|  | Hs_CHRNB2 |  | P17787 | Homo sapiens |
|  | Hs_CHRNB3 |  | Q05901 | Homo sapiens |
|  | Hs_CHRNB4 |  | P30926 | Homo sapiens |
|  | Hs_CHRND |  | Q07001 | Homo sapiens |
|  | Hs_CHRNE |  | Q04844 | Homo sapiens |
|  | Hs_CHRNG |  | P07510 | Homo sapiens |
|  |  |  |  |  |
|  |  |  |  |  |
| **Fig S8** | Dm_CG11340 |  | Q9V9Y4 | Drosophila melanogaster |
|  | Dm_CG12344 |  | Q8SWZ1 | Drosophila melanogaster |
|  | Dm_CG6927 |  | Q9W4G1 | Drosophila melanogaster |
|  | Dm_CG7589 |  | Q9VVH4 | Drosophila melanogaster |
|  | Dm_CG8916 |  | Q9VXL9 | Drosophila melanogaster |
|  | Dm_GluCl |  | Q94900 | Drosophila melanogaster |
|  | Dm_Grd |  | Q24352 | Drosophila melanogaster |
|  | Dm_hclA |  | Q9VDU9 | Drosophila melanogaster |
|  | Dm_hclB |  | B5SUY9 | Drosophila melanogaster |
|  | Dm_Lcch3 |  | Q08832 | Drosophila melanogaster |
|  | Dm_pHCl |  | Q2PDZ0 | Drosophila melanogaster |
|  | Dm_Rdl |  | P25123 | Drosophila melanogaster |
|  | Dpul_8916 |  | NCBI_GNO_810024 | Daphnia pulex |
|  | Dpul_GluCl |  | NCBI_GNO_164584 | Daphnia pulex |
|  | Dpul_Grd |  | NCBI_GNO_408024 | Daphnia pulex |
|  | Dpul_hclA |  | NCBI_GNO_254493 | Daphnia pulex |
|  | Dpul_hclB |  | NCBI_GNO_18654 | Daphnia pulex |
|  | Dpul_Lcch3 |  | NCBI_GNO_654024 | Daphnia pulex |
|  | Dpul_Rdl |  | NCBI_GNO_104424 | Daphnia pulex |
|  | Dpul_RdlL |  | NCBI_GNO_144104 | Daphnia pulex |
|  | Hs_GABRA1 |  | P14867 | Homo sapiens |
|  | Hs_GABRA2 |  | P47869 | Homo sapiens |
|  | Hs_GABRA3 |  | P34903 | Homo sapiens |
|  | Hs_GABRA4 |  | P48169 | Homo sapiens |
|  | Hs_GABRA5 |  | P31644 | Homo sapiens |
|  | Hs_GABRA6 |  | Q16445 | Homo sapiens |
|  | Hs_GABRB1 |  | P18505 | Homo sapiens |
|  | Hs_GABAB2 |  | P47870 | Homo sapiens |
|  | Hs_GABRB3 |  | P28472 | Homo sapiens |
|  | Hs_GABRD |  | O14764 | Homo sapiens |
|  | Hs_GABRE |  | P78334 | Homo sapiens |
|  | Hs_GABRG1 |  | Q8N1C3 | Homo sapiens |
|  | Hs_GABRG2 |  | P18507 | Homo sapiens |
|  | Hs_GABRG3 |  | Q99928 | Homo sapiens |
|  | Hs_GABRP |  | O00591 | Homo sapiens |
|  | Hs_GABRQ |  | Q9UN88 | Homo sapiens |
|  | Hs_GABRR1 |  | P24046 | Homo sapiens |
|  | Hs_GABRR2 |  | P28476 | Homo sapiens |
|  | Hs_GABRR3 |  | A8MPY1 | Homo sapiens |
|  | Hs_GLRA1 |  | P23415 | Homo sapiens |
|  | Hs_GLRA2 |  | P23416 | Homo sapiens |
|  | Hs_GLRA3 |  | O75311 | Homo sapiens |
|  | Hs_GLRA4 |  | Q5JXX5 | Homo sapiens |
|  | Hs_GLRB |  | P48167 | Homo sapiens |
|  | Hs_HTR3A | 5HT3R | P46098 | Homo sapiens |
|  | Hs_HTR3B |  | O95264 | Homo sapiens |
|  | Hs_HTR3C |  | Q8WXA8 | Homo sapiens |
|  | Hs_HTR3D |  | Q70Z44 | Homo sapiens |
|  | Hs_HTR3E |  | A5X5Y0 | Homo sapiens |
|  | Hs_ZACN |  | Q401N2 | Homo sapiens |
|  |  |  |  |  |
|  |  |  |  |  |
| **Fig S9** | Ce_acd-1 | C24G7.2 | P91102 | Caenorhabditis elegans |
|  | Ce_acd-2 | C24G7.4 | P91100 | Caenorhabditis elegans |
|  | Ce_acd-4 | F28A12.1 | Q22970 | Caenorhabditis elegans |
|  | Ce_asic-1 | ZK770.1 | O01635 | Caenorhabditis elegans |
|  | Ce_asic-2 | T28F4.2 | Q22851 | Caenorhabditis elegans |
|  | Ce_C24G7.1 |  | P91103 | Caenorhabditis elegans |
|  | Ce_deg-1 |  | P24585 | Caenorhabditis elegans |
|  | Ce_del-1 |  | Q19038 | Caenorhabditis elegans |
|  | Ce_del-9 | C18B2.6 | Q18077 | Caenorhabditis elegans |
|  | Ce_F23B2.3 |  | O45402 | Caenorhabditis elegans |
|  | Ce_flr-1 |  | G5EGI5 | Caenorhabditis elegans |
|  | Ce_mec-4 |  | P24612 | Caenorhabditis elegans |
|  | Ce_mec-10 |  | P34886 | Caenorhabditis elegans |
|  | Ce_UNC-8 |  | Q21974 | Caenorhabditis elegans |
|  | Ce_UNC-105 |  | Q09274 | Caenorhabditis elegans |
|  | Dm_ppk |  | O44940 | Drosophila melanogaster |
|  | Dm_ppk4 |  | O61365 | Drosophila melanogaster |
|  | Dm_ppk6 |  | Q86LH3 | Drosophila melanogaster |
|  | Dm_ppk7 |  | Q9VME9 | Drosophila melanogaster |
|  | Dm_ppk10 |  | Q86LH1 | Drosophila melanogaster |
|  | Dm_ppk11 |  | Q9VL84 | Drosophila melanogaster |
|  | Dm_ppk12 |  | Q9W250 | Drosophila melanogaster |
|  | Dm_ppk13 |  | Q86LG9 | Drosophila melanogaster |
|  | Dm_ppk14 |  | Q9VME8 | Drosophila melanogaster |
|  | Dm_ppk16 |  | Q86LG7 | Drosophila melanogaster |
|  | Dm_ppk19 |  | Q9VAJ3 | Drosophila melanogaster |
|  | Dm_ppk20 |  | Q9VAJ6 | Drosophila melanogaster |
|  | Dm_ppk21 |  | Q9VAJ7 | Drosophila melanogaster |
|  | Dm_ppk23 |  | Q86LG3 | Drosophila melanogaster |
|  | Dm_ppk25 |  | A1Z6S4 | Drosophila melanogaster |
|  | Dm_ppk28 |  | Q86LG1 | Drosophila melanogaster |
|  | Dm_rpk |  | O46342 | Drosophila melanogaster |
|  | Dpul_DEG/ENaC-1 |  | JGI_V11_94123 | Daphnia pulex |
|  | Dpul_DEG/ENaC-2 |  | NCBI_GNO_300334 | Daphnia pulex |
|  | Dpul_DEG/ENaC-3 |  | NCBI_GNO_630374 | Daphnia pulex |
|  | Dpul_DEG/ENaC-4 |  | NCBI_GNO_708174 | Daphnia pulex |
|  | Dpul_DEG/ENaC-5 |  | NCBI_GNO_413584 | Daphnia pulex |
|  | Dpul_DEG/ENaC-6 |  | NCBI_GNO_400244 | Daphnia pulex |
|  | Dpul_DEG/ENaC-7 |  | NCBI_GNO_538084 | Daphnia pulex |
|  | Dpul_DEG/ENaC-8 |  | NCBI_GNO_188894 | Daphnia pulex |
|  | Dpul_DEG/ENaC-9 |  | NCBI_GNO_550033 | Daphnia pulex |
|  | Dpul_DEG/ENaC-10 |  | NCBI_GNO_532034 | Daphnia pulex |
|  | Dpul_DEG/ENaC-11 |  | NCBI_GNO_530034 | Daphnia pulex |
|  | Dpul_DEG/ENaC-12 |  | NCBI_GNO_534034 | Daphnia pulex |
|  | Dpul_DEG/ENaC-13 |  | NCBI_GNO_536034 | Daphnia pulex |
|  | Dpul_DEG/ENaC-14 |  | JGI_V11_98455 | Daphnia pulex |
|  | Hs_ASIC1 |  | P78348 | Homo sapiens |
|  | Hs_ASIC2 |  | Q16515 | Homo sapiens |
|  | Hs_ASIC3 |  | Q9UHC3 | Homo sapiens |
|  | Hs_ASIC4 |  | Q96FT7 | Homo sapiens |
|  | Hs_SCNN1A |  | P37088 | Homo sapiens |
|  | Hs_SCNN1B |  | P51168 | Homo sapiens |
|  | Hs_SCNN1D |  | P51172 | Homo sapiens |
|  | Hs_SCNN1G |  | P51170 | Homo sapiens |
|  |  |  |  |  |
|  |  |  |  |  |
| **Fig S10-A** | Dd_P2X1 |  | Q86JM7 | Dictyostelium discoideum |
|  | Dd_P2X2 |  | Q553Y1 | Dictyostelium discoideum |
|  | Dd_P2X3 |  | Q553Y0 | Dictyostelium discoideum |
|  | Dd_P2X4 |  | Q54J33 | Dictyostelium discoideum |
|  | Dd_P2X5 |  | Q54JH4 | Dictyostelium discoideum |
|  | Dpul_P2XL1 |  | NCBI_GNO_488034 | Daphnia pulex |
|  | Dpul_P2XL2 |  | NCBI_GNO_490034 | Daphnia pulex |
|  | Hs_P2RX1 |  | P51575 | Homo sapiens |
|  | Hs_P2RX2 |  | Q9UBL9 | Homo sapiens |
|  | Hs_P2RX3 |  | P56373 | Homo sapiens |
|  | Hs_P2RX4 |  | Q99571 | Homo sapiens |
|  | Hs_P2RX5 |  | Q93086 | Homo sapiens |
|  | Hs_P2RX6 |  | O15547 | Homo sapiens |
|  | Hs_P2RX7 |  | Q99572 | Homo sapiens |
|  |  |  |  |  |
|  |  |  |  |  |
| **Fig S10-B** | Ce_ORAI |  | Q09232 | Caenorhabditis elegans |
|  | Dm_ORAI |  | Q9U6B8 | Drosophila melanogaster |
|  | Dpul_ORAI |  | NCBI_GNO_352053 | Daphnia pulex |
|  | Hs_ORAI1 | CRACM1 | Q96D31 | Homo sapiens |
|  | Hs_ORAI2 | CRACM2 | Q8BH10 | Homo sapiens |
|  | Hs_ORAI3 | CRACM3 | Q9BRQ5 | Homo sapiens |
|  |  |  |  |  |
|  |  |  |  |  |
| **Fig S10-C** | Ce_IP3R |  | Q9U3B3 | Caenorhabditis elegans |
|  | Dm_IP3R |  | P29993 | Drosophila melanogaster |
|  | Dpul_IP3R |  | NCBI_GNO_672594 | Daphnia pulex |
|  | Hs_IP3R2 |  | Q14571 | Homo sapiens |
|  | Hs_IP3R3 |  | Q14573 | Homo sapiens |
|  | Hs_IP3R1 |  | Q14643 | Homo sapiens |
|  |  |  |  |  |
|  |  |  |  |  |
| **Fig S10-D** | Ce_RYR |  | P91905 | Caenorhabditis elegans |
|  | Dm_RyR |  | Q24498 | Drosophila melanogaster |
|  | Dpul_RYR |  | NCBI_GNO_1700023 | Daphnia pulex |
|  | Hs_RYR1 |  | P21817 | Homo sapiens |
|  | Hs_RYR2 |  | Q92736 | Homo sapiens |
|  | Hs_RYR3 |  | Q15413 | Homo sapiens |
|  |  |  |  |  |
|  |  |  |  |  |
| **Fig S11-A** | Ce_clh-1 |  | G5EDQ0 | Caenorhabditis elegans |
|  | Ce_clh-2 |  | G5EGQ0 | Caenorhabditis elegans |
|  | Ce_clh-3 |  | Q7KKH7 | Caenorhabditis elegans |
|  | Ce_clh-4 |  | Q9U6W5 | Caenorhabditis elegans |
|  | Ce_clh-5 |  | Q9U6W4 | Caenorhabditis elegans |
|  | Ce_clh-6 |  | G5EC75 | Caenorhabditis elegans |
|  | Dm_ClC-a |  | Q9VGH7 | Drosophila melanogaster |
|  | Dm_ClC-b |  | Q7JZ25 | Drosophila melanogaster |
|  | Dm_ClC-c |  | Q8MQK2 | Drosophila melanogaster |
|  | Dpul_CLC-a1 |  | NCBI_GNO_414034 | Daphnia pulex |
|  | Dpul_CLC-a2 |  | NCBI_GNO_418034 | Daphnia pulex |
|  | Dpul_CLC-a3 |  | NCBI_GNO_420034 | Daphnia pulex |
|  | Dpul_CLC-a4 |  | NCBI_GNO_422034 | Daphnia pulex |
|  | Dpul_CLC-b |  | NCBI_GNO_38903 | Daphnia pulex |
|  | Dpul_CLC-c1 |  | NCBI_GNO_206284 | Daphnia pulex |
|  | Dpul_CLC-c2 |  | NCBI_GNO_338114 | Daphnia pulex |
|  | Hs_CLCN1 |  | P35523 | Homo sapiens |
|  | Hs_CLCN2 |  | P51788 | Homo sapiens |
|  | Hs_CLCN3 |  | P51790 | Homo sapiens |
|  | Hs_CLCN4 |  | P51793 | Homo sapiens |
|  | Hs_CLCN5 |  | P51795 | Homo sapiens |
|  | Hs_CLCN6 |  | P51797 | Homo sapiens |
|  | Hs_CLCN7 |  | H0Y2M6 | Homo sapiens |
|  | Hs_CLCNKA |  | P51800 | Homo sapiens |
|  | Hs_CLCNKB |  | P51801 | Homo sapiens |
|  |  |  |  |  |
|  |  |  |  |  |
| **Fig S11-B** | Ce_exc-4 |  | Q8WQA4 | Caenorhabditis elegans |
|  | Ce_exl-1 |  | O45405 | Caenorhabditis elegans |
|  | Dm_Clic |  | Q9VY78 | Drosophila melanogaster |
|  | Dpul_Clic |  | NCBI_GNO_772014 | Drosophila melanogaster |
|  | Dpul_Clic-like |  | NCBI_GNO_2066033 | Daphnia pulex |
|  | Hs_CLIC1 |  | O00299 | Homo sapiens |
|  | Hs_CLIC2 |  | E9PGQ2 | Homo sapiens |
|  | Hs_CLIC3 |  | O95833 | Homo sapiens |
|  | Hs_CLIC4 |  | Q9Y696 | Homo sapiens |
|  | Hs_CLIC5 |  | Q9NZA1 | Homo sapiens |
|  | Hs_CLIC6 |  | Q96NY7 | Homo sapiens |
|  |  |  |  |  |
|  |  |  |  |  |
| **Fig S11-C** | Ce_B0564.3 |  | Q17528 | Caenorhabditis elegans |
|  | Ce_BEST1 |  | Q21973 | Caenorhabditis elegans |
|  | Ce_T19C3.1 |  | Q22566 | Caenorhabditis elegans |
|  | Ce_T20G5.4 |  | P34577 | Caenorhabditis elegans |
|  | Ce_ZC518.1 |  | Q23369 | Caenorhabditis elegans |
|  | Ce_ZK688.2 |  | P34672 | Caenorhabditis elegans |
|  | Dm_Best1 |  | Q9V3J6 | Drosophila melanogaster |
|  | Dm_Best2 |  | Q9VRW4 | Drosophila melanogaster |
|  | Dm_Best3 |  | Q9VUM7 | Drosophila melanogaster |
|  | Dm_Best4 |  | Q9VUM6 | Drosophila melanogaster |
|  | Dpul_Best1 |  | NCBI_GNO_298194 | Daphnia pulex |
|  | Dpul_Best2 |  | NCBI_GNO_588594 | Daphnia pulex |
|  | Dpul_Best3 |  | NCBI_GNO_620014 | Daphnia pulex |
|  | Dpul_Best4 |  | NCBI_GNO_1176044 | Daphnia pulex |
|  | Hs_BEST1 |  | O76090 | Homo sapiens |
|  | Hs_BEST2 |  | Q8NFU1 | Homo sapiens |
|  | Hs_BEST3 |  | Q8N1M1 | Homo sapiens |
|  | Hs_BEST4 |  | Q8NFU0 | Homo sapiens |
|  |  |  |  |  |
|  |  |  |  |  |
| **Fig S11-D** | Ce_ttyh-1 |  | Q20332 | Caenorhabditis elegans |
|  | Dm_CG3638 |  | Q9W5A5 | Drosophila melanogaster |
|  | Dm_tty |  | Q9U6L4 | Drosophila melanogaster |
|  | Dpul_tty |  | NCBI_GNO_242114 | Daphnia pulex |
|  | Hs_TTYH1 |  | Q9H313 | Homo sapiens |
|  | Hs_TTYH2 |  | Q9BSA4 | Homo sapiens |
|  | Hs_TTYH3 |  | Q9C0H2 | Homo sapiens |
|  |  |  |  |  |
|  |  |  |  |  |
| **Fig S11-E** | Ce_anoh-1 |  | C0P286 | Caenorhabditis elegans |
|  | Ce_anoh-2 |  | Q8I4C3 | Caenorhabditis elegans |
|  | Dm_Axs |  | O97132 | Drosophila melanogaster |
|  | Dm_CG10353 |  | Q8MT62 | Drosophila melanogaster |
|  | Dm_CG15270 |  | Q9V416 | Drosophila melanogaster |
|  | Dm_CG16718 |  | Q86P24 | Drosophila melanogaster |
|  | Dm_CG6938 |  | Q9VTS0 | Drosophila melanogaster |
|  | Dpul_ANO1 |  | NCBI_GNO_736094 | Daphnia pulex |
|  | Dpul_ANO2 |  | NCBI_GNO_408014 | Daphnia pulex |
|  | Dpul_ANO3 |  | NCBI_GNO_694034 | Daphnia pulex |
|  | Hs_ANO1 | TMEM16A | Q5XXA6 | Homo sapiens |
|  | Hs_ANO2 | TMEM16B | Q9NQ90 | Homo sapiens |
|  | Hs_ANO3 | TMEM16C | Q9BYT9 | Homo sapiens |
|  | Hs_ANO4 | TMEM16D | Q32M45 | Homo sapiens |
|  | Hs_ANO5 | TMEM16E | Q75V66 | Homo sapiens |
|  | Hs_ANO6 | TMEM16F | Q4KMQ2 | Homo sapiens |
|  | Hs_ANO7 | TMEM16G | Q6IWH7 | Homo sapiens |
|  | Hs_ANO8 | TMEM16H | Q9HCE9 | Homo sapiens |
|  | Hs_ANO9 | TMEM16J | A1A5B4 | Homo sapiens |
|  | Hs_ANO10 | TMEM16K | Q9NW15 | Homo sapiens |
