# Supplementary figures and images for: Effects of midazolam, pentobarbital and ketamine on the mRNA expression of ion channels in a model organism Daphnia pulex
Source: BMC Anesthesiol. 2013 Oct 18;13:32. doi: 10.1186/1471-2253-13-32 (PMC3879215; doi:10.1186/1471-2253-13-32)

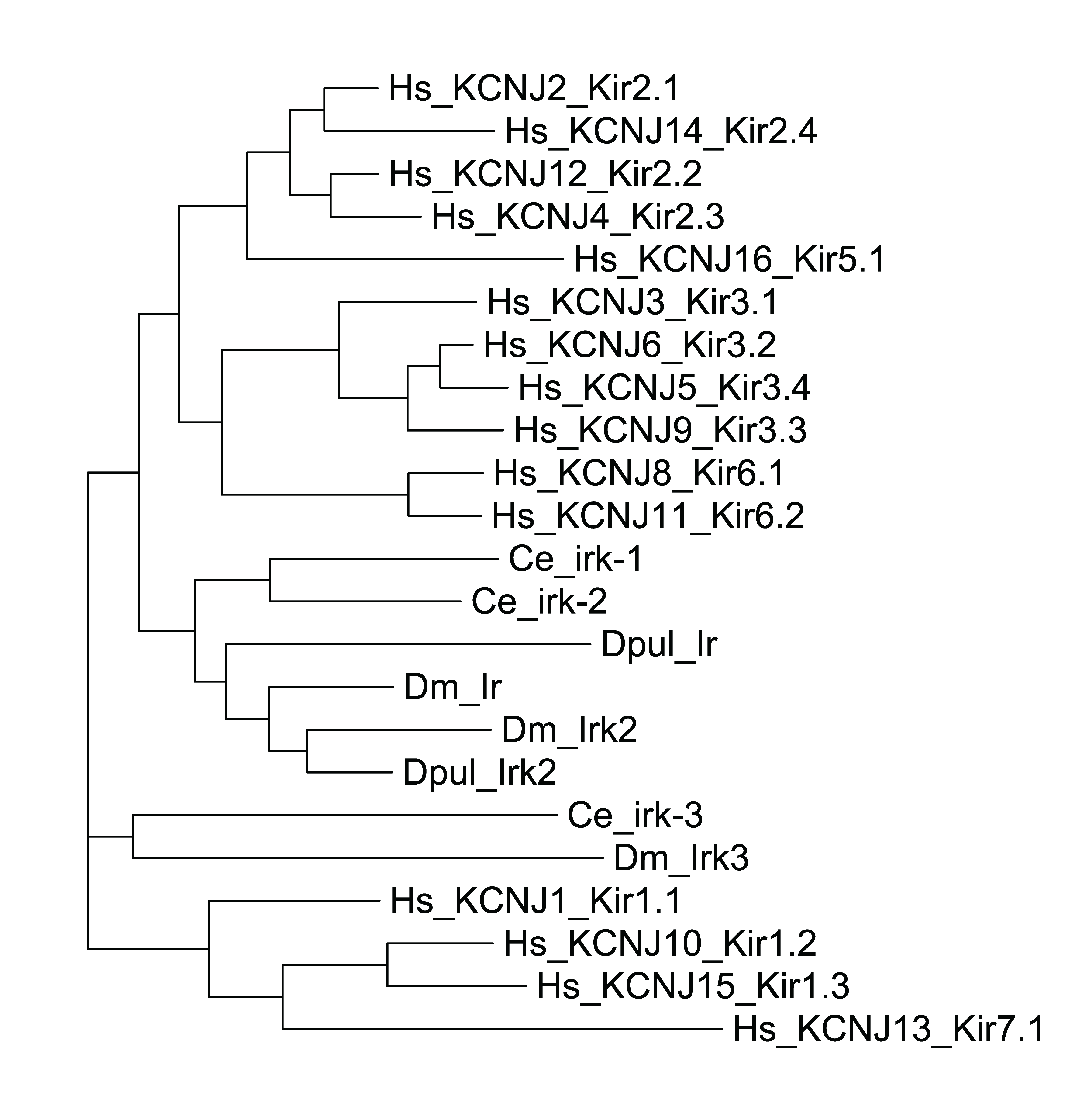

Supplement: Additional file 3: Figure S1 — Phylogenetic tree of the 2TM-KV (Kir) channels. The Kir subunit possesses a P-domain composed of two transmembrane helices connected by a P-loop. Four Kir subunits assemble to for a channel. The mammalian Kir family comprises the classical inward-rectifying K+ channels (Kir2), the KATP channels (Kir6), G protein-activated K+ channels (Kir3), and K+-transport channels. The invertebrate Kir genes form distinct clusters. The Daphnia genome contains two Kir genes: Dpul_Ir and Dpul_Irk2. The homolog of the Drosophila Irk3 is absent in Daphnia genome. Abbreviation: Hs, Homo sapiens; Dm, Drosophila melanogaster; Ce, Caenorhabditis elegans; Dpul, Daphnia pulex. [file 1471-2253-13-32-S3.tiff]

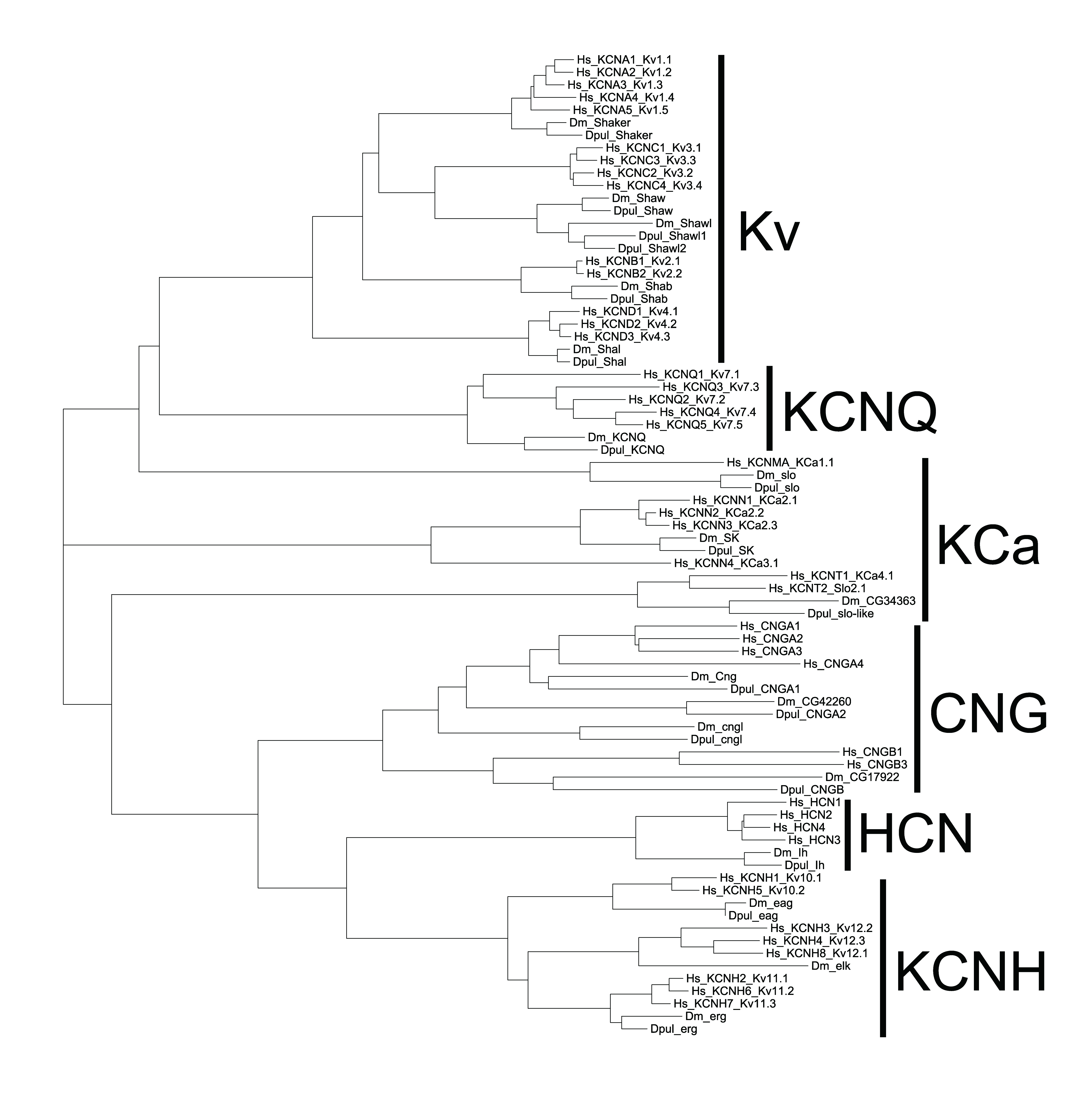

Supplement: Additional file 4: Figure S2 — Phylogenetic tree of the 6TM-Kv, CNG and HCN channels. The 6TM-Kv channels are highly diverse in sequence, structure and function. In addition to the P-domain, each 6TM-KV subunit obtains a voltage sensor domain (VSD) composed of four transmembrane helices. The CNG and HCN channels, although not classified as Kv channels, are homologous to the KCNH KV channels. Daphnia and Drosophila share similar gene sets for the 6TM-KV/CNG/HCN channel group, with subtle differences. The homolog of the Drosophila elk is absent in the Daphnia genome. Daphnia have two homologs of the Drosophila Shawl genes, namely Shawl1 and Shawl2, while Drosophila have one. [file 1471-2253-13-32-S4.tiff]

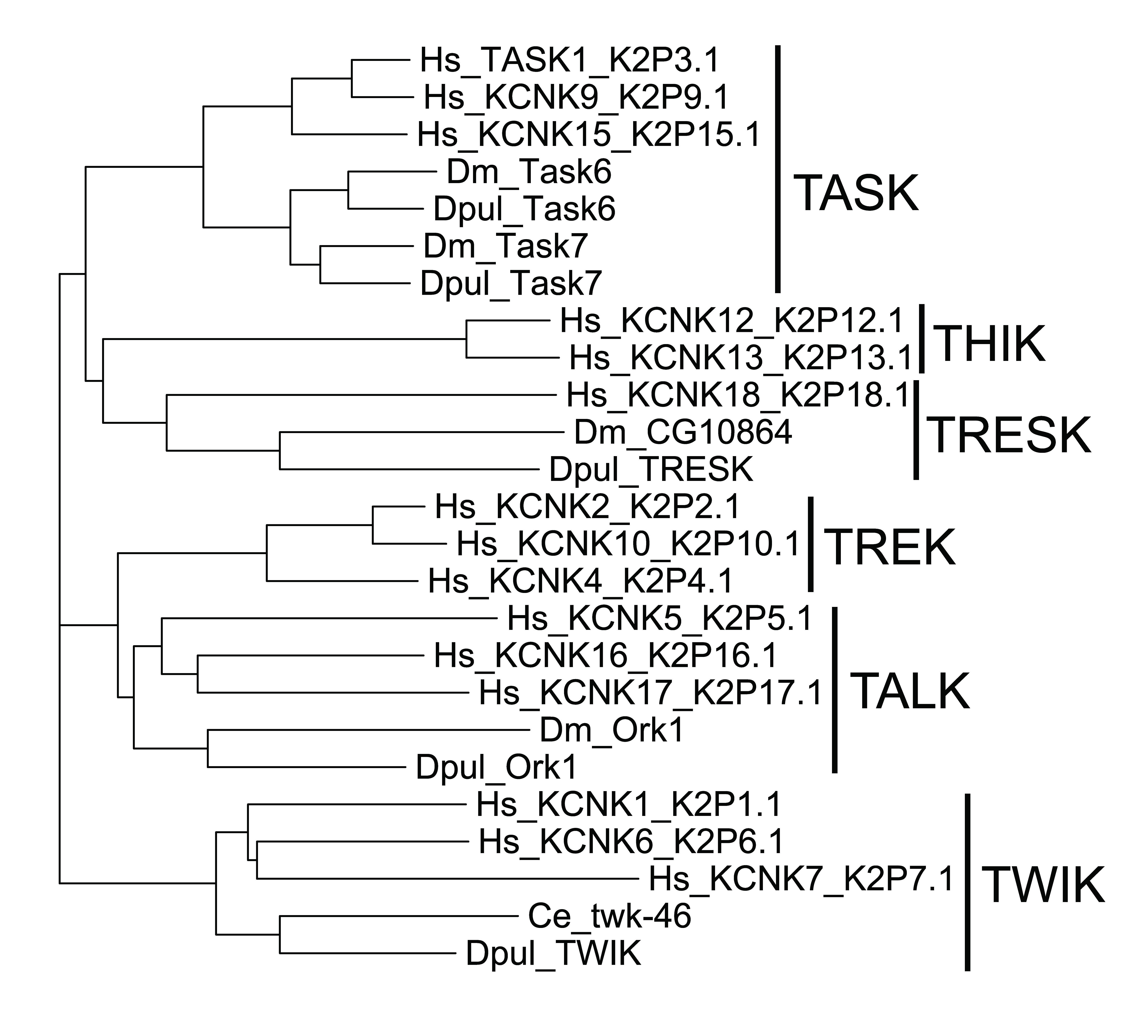

Supplement: Additional file 5: Figure S3 — Phylogenetic tree of the 4TM-KV (K2P) channels. The K2P subunit consists of two P-domains, and two such subunits assemble to form a channel. Daphnia have five K2P members: Dpul_TWIK, Dpul_Task6, Dpul_Task7, Dpul_Ork1 and Dpul_TRESK. The Daphnia homolog of the mammalian TWIK is detected at transcript level, but the Drosophila counterpart is absent. As in Drosophila, the TREK member of the K2P channels is also absent in Daphnia. Abbreviation: Hs, Homo sapiens; Dm, Drosophila melanogaster; Ce, Caenorhabditis elegans; Dpul, Daphnia pulex. [file 1471-2253-13-32-S5.tiff]

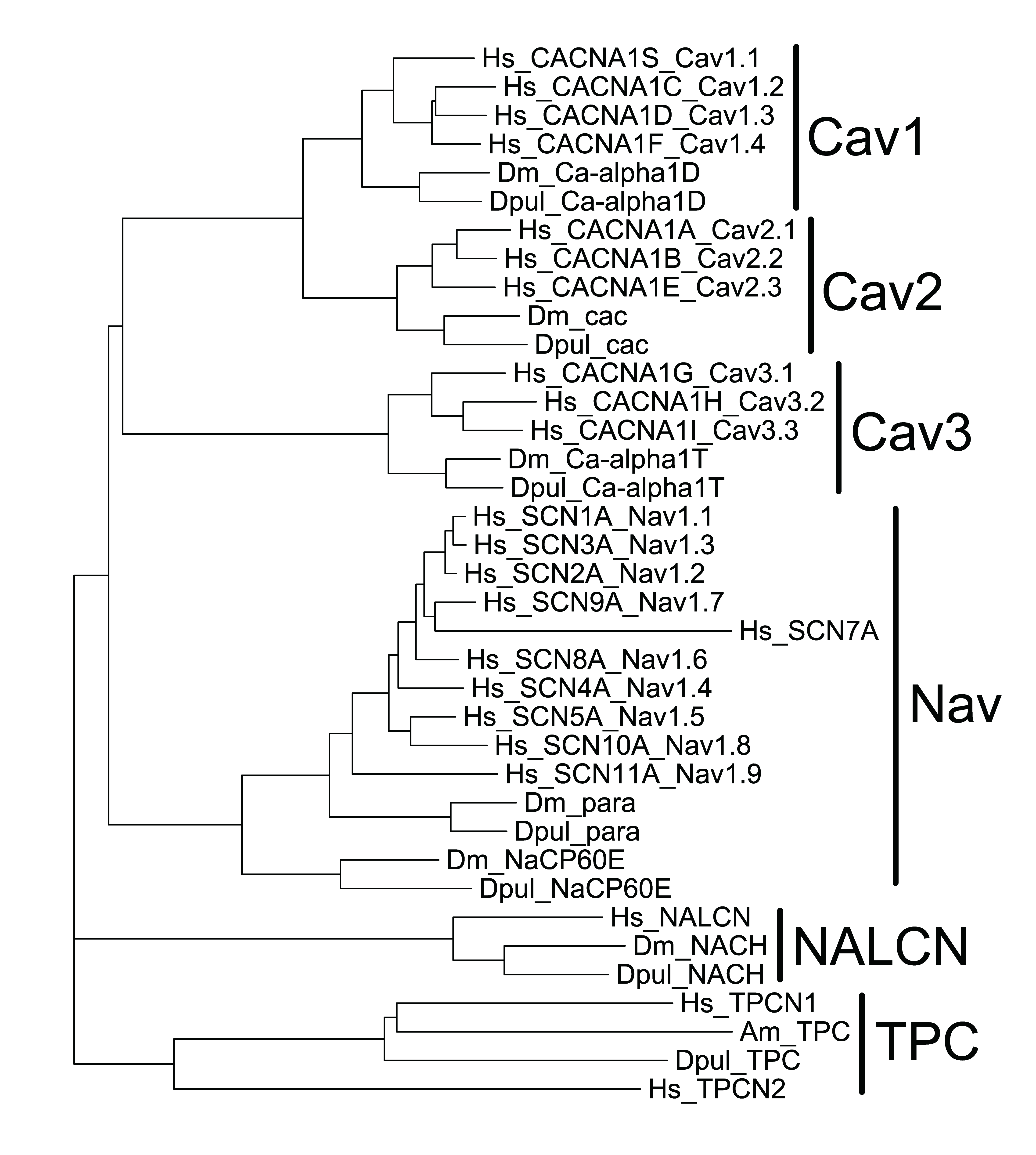

Supplement: Additional file 6: Figure S4 — Phylogenetic tree of the CaV, NaV, NALCN and TPC channels. The CaV and NaV channels are thought to arise from the potassium channels during evolution. The CaV/NaV channel contains a single principal (α1) subunit with four 6TM domains. The human genome contains ten α1 genes, which can be further clustered in three groups: CaV1 (L-type), CaV2 (P/Q, N, and R type), and CaV3 (T-type). The Daphnia genome predicts three genes: Dpul_Ca-alpha1D (CaV1), Dpul_Ca-alpha1T (CaV2) and Dpul_cac (CaV3). NALCN is represented by a single gene in human, Daphnia and Drosophila. Unlike the CaV, NaV and NALCN, the TPC subunit contains two P-domains and assembles as a dimer. The transcript of the TPC encoding gene is detected in Daphnia. TPC Gene models are also available for many insects, such as Apis mellifera, but absent in Drosophila. Abbreviation: Hs, Homo sapiens; Dm, Drosophila melanogaster; Am, Apis mellifera; Caenorhabditis elegans; Dpul, Daphnia pulex. [file 1471-2253-13-32-S6.tiff]

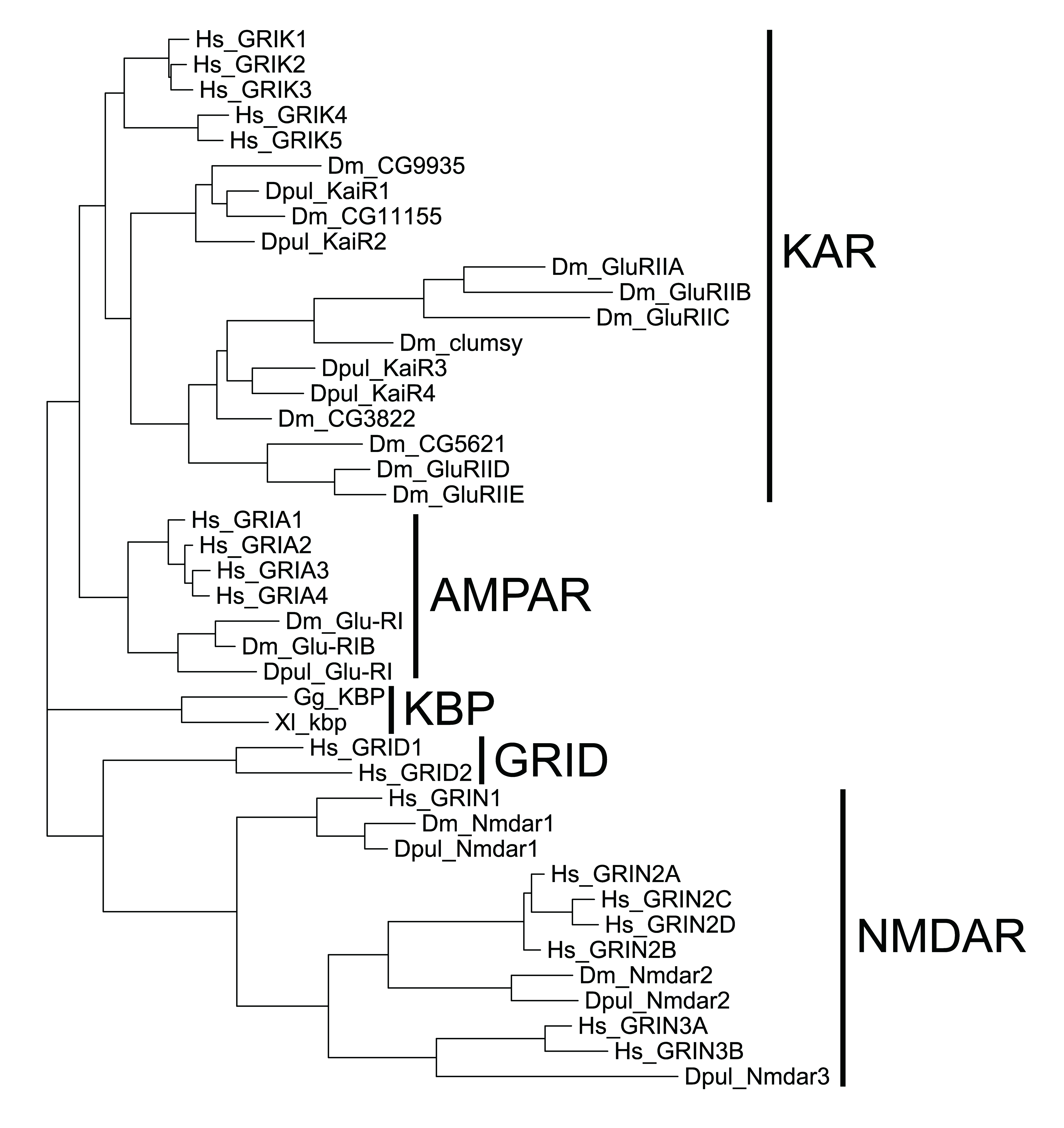

Supplement: Additional file 8: Figure S6 — Phylogenetic tree of the iGluRs. The iGluR is a heterotetramer containing four P-domains, each in one subunit. The mammalian iGluRs are divided into three groups: AMPA, NMDA and KA receptors. There are two other classes with respect to sequence similarity known as the delta class (GRID) and the kainate-binding proteins (KBP). The invertebrate iGluRs display considerable variations, especially in the KAR branch. In addition, a GRIN3-like gene is detected in Daphnia at the transcript level, but it is absent in Drosophila. Abbreviation: Hs, Homo sapiens; Dm, Drosophila melanogaster; Xl, Xenopus laevis; Gg, Gallus gallus; Dpul, Daphnia pulex. [file 1471-2253-13-32-S8.tiff]

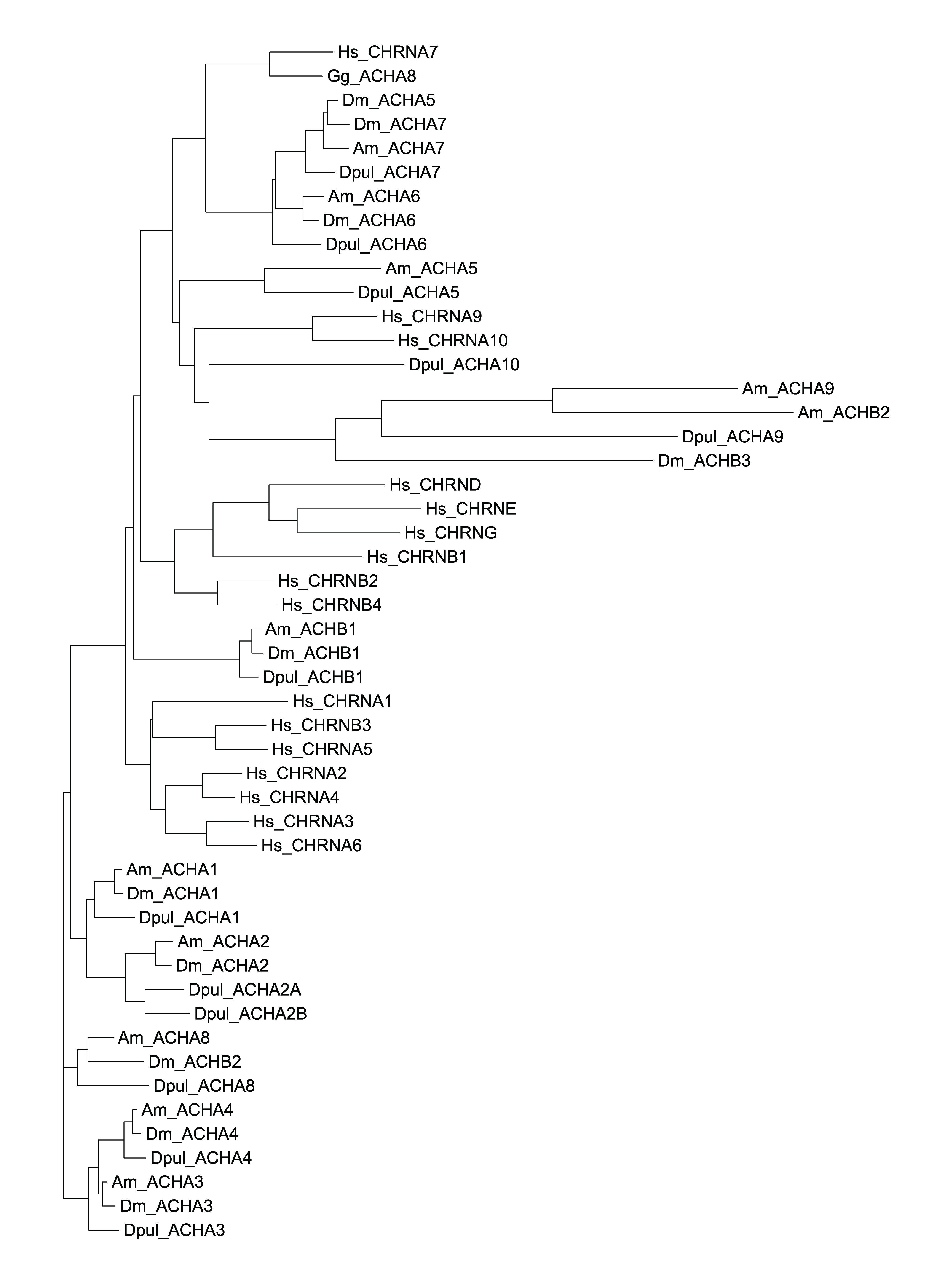

Supplement: Additional file 9: Figure S7 — Phylogenetic tree of the nAChRs. The nAChRs belong to the Cys-loop receptor superfamily. The nAChRs are highly diverse in both vertebrates and invertebrates. Daphnia genome encodes 12 putative nAChR genes. Abbreviation: Hs, Homo sapiens; Dm, Drosophila melanogaster; Gg, Gallus gallus; Am, Apis mellifera; Dpul, Daphnia pulex. [file 1471-2253-13-32-S9.tiff]

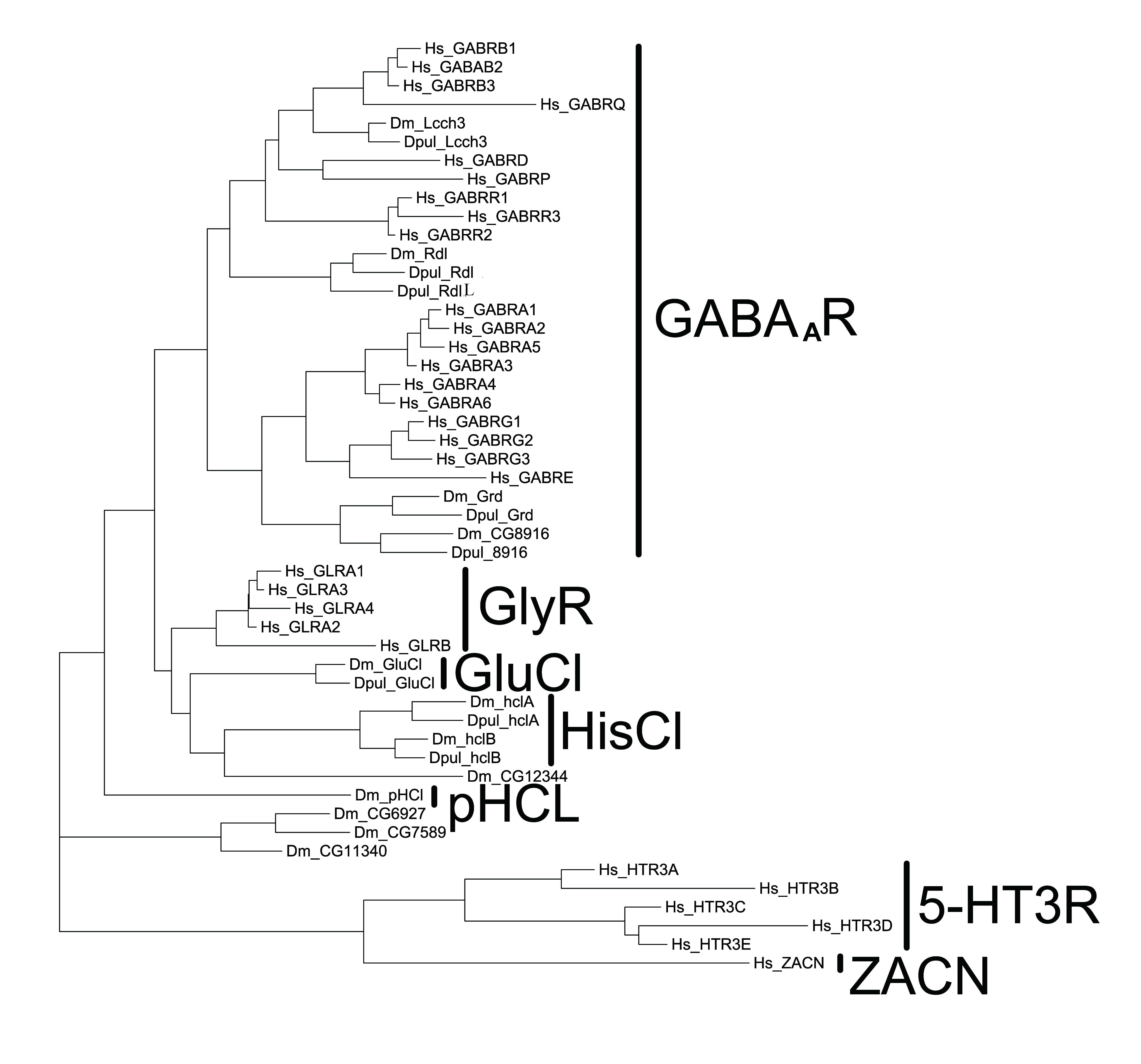

Supplement: Additional file 10: Figure S8 — Phylogenetic tree of non-nAChR cys-loop receptors. In addition to the nAChRs, the Cys-loop receptor superfamily also includes 5-hydroxytryptamine type 3 receptors (5-HT3Rs), zinc-activated ion channels (ZACNs), GABAARs and glycine receptors (GlyRs). The Daphnia genome lacks the counterparts of the Drosophila genes CG11340, CG6927 and CG7589, which form a separate branch. The pHCl gene seen in Drosophila is also absent in Daphnia. [file 1471-2253-13-32-S10.tiff]

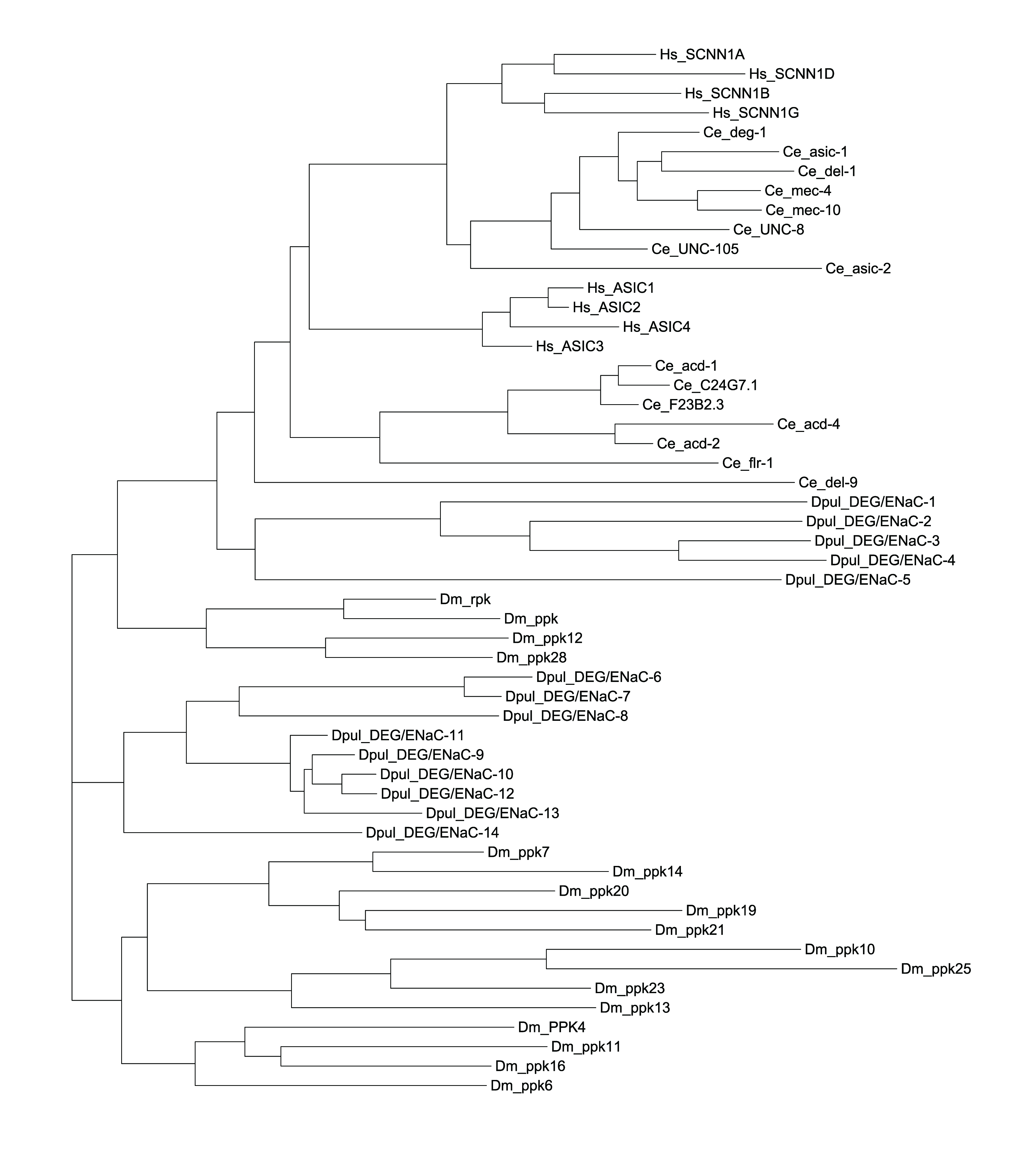

Supplement: Additional file 11: Figure S9 — Phylogenetic tree of the Deg/ENaC channels. DEG/ENaC channels have been found in nematodes, insects and vertebrates and implicated in a broad spectrum of cellular functions. Mammalian DEG/ENaC channels fall into two major groups (EnaC and ASIC) with 9 members. The invertebrate DEG/ENaC members are highly diverse. Fourteen Daphnia ENaC/Deg homologs are detected at transcript level. [file 1471-2253-13-32-S11.tiff]

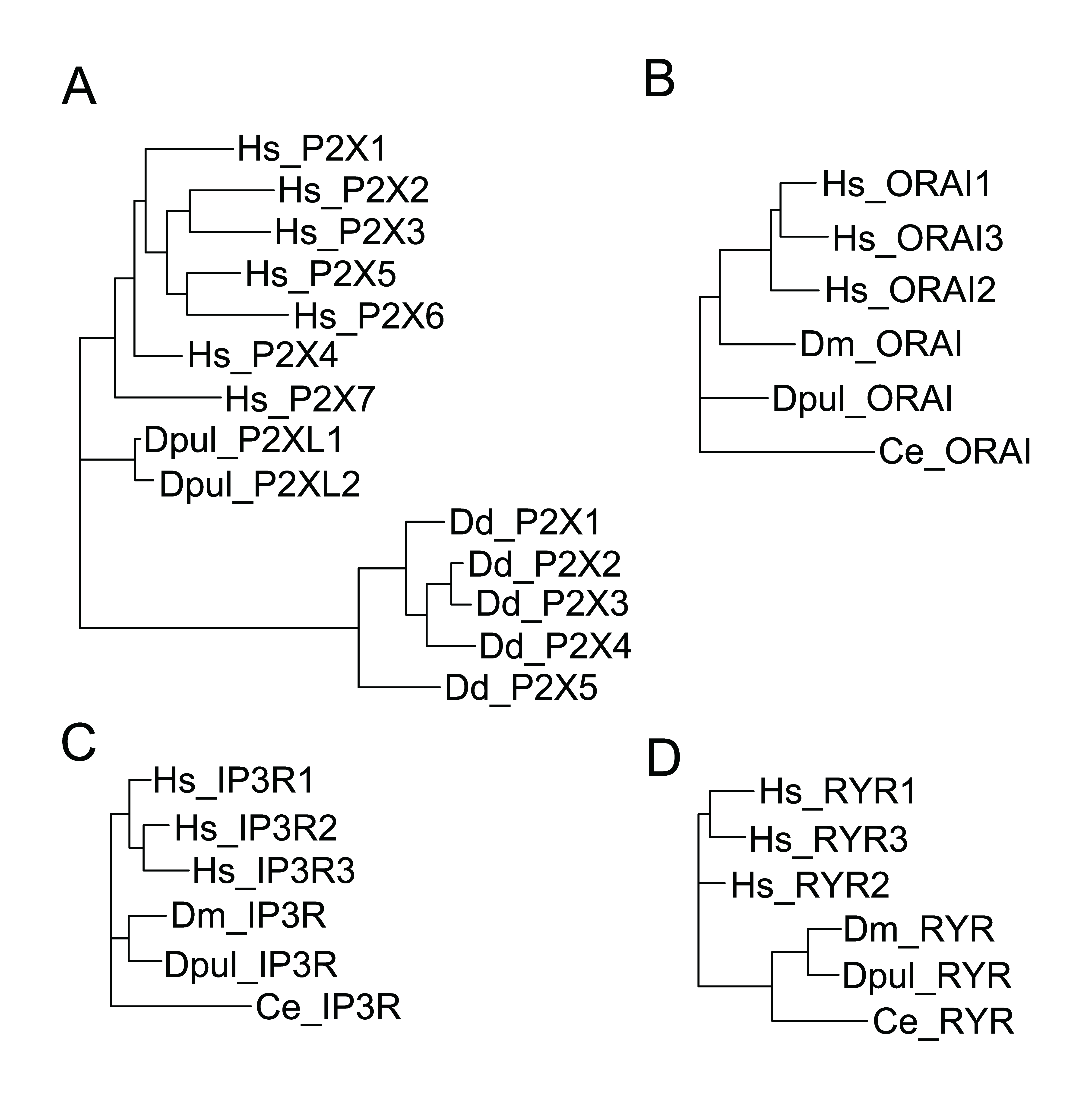

Supplement: Additional file 12: Figure S10 — Phylogenetic trees of the P2X receptor, ORAI proteins, IP3Rs and RyRs. Two P2X gene transcripts are detected in Daphnia, meanwhile, one ORAI, one IP3R and one RyR are detected in Daphnia. [file 1471-2253-13-32-S12.tiff]
